# Major chromosome rearrangements in intergeneric wheat × rye hybrids in compatible and incompatible crosses detected by GBS read coverage analysis

**DOI:** 10.1038/s41598-024-61622-1

**Published:** 2024-05-14

**Authors:** Natalia Tikhenko, Max Haupt, Jörg Fuchs, Dragan Perovic, Axel Himmelbach, Martin Mascher, Andreas Houben, Twan Rutten, Manuela Nagel, Natalia V. Tsvetkova, Stefanie Sehmisch, Andreas Börner

**Affiliations:** 1 ROR (Research Organization Registry), Leibniz Institute of Plant Genetics and Crop Plant Research (IPK), Corrensstr 3, 06466 OT Gatersleben, Seeland, Germany; 2 Federal Research Centre for Cultivated Plants, Institute for Resistance Research and Stress Tolerance, Julius Kuehn Institute, Erwin-Baur Strasse 27, 06484 Quedlinburg, Germany; 3 German Centre for Integrative Biodiversity Research (iDiv) Halle-Jena-Leipzig, Leipzig, Germany; 4 Saint-Petersburg State University (SPbSU), St. Petersburg, 199034 Russia; 5 Vavilov Institute of General Genetics Russian Academy of Sciences, Moscow, 119991 Russia; 6 Institute of Agricultural and Nutritional Sciences, Martin Luther University Halle‐Wittenberg, Betty‐Heimann‐Straße 3, 06120 Halle, Germany

**Keywords:** Wheat-rye amphidiploids, Incompatible crosses, Chromosome rearrangements, Embryo lethality, Hybrid sterility, Genotyping-by-sequencing GBS, Coverage analysis, Genetics, Molecular biology

## Abstract

The presence of incompatibility alleles in primary amphidiploids constitutes a reproductive barrier in newly synthesized wheat-rye hybrids. To overcome this barrier, the genome stabilization process includes large-scale chromosome rearrangements. In incompatible crosses resulting in fertile amphidiploids, the elimination of one of the incompatible alleles *Eml-A1* or *Eml-R1b* can occur already in the somatic tissue of the wheat × rye hybrid embryo. We observed that the interaction of incompatible loci *Eml-A1* of wheat and *Eml-R1b* of rye after overcoming embryo lethality leads to hybrid sterility in primary triticale. During subsequent seed reproductions (R_1_, R_2_ or R_3_) most of the chromosomes of A, B, D and R subgenomes undergo rearrangement or eliminations to increase the fertility of the amphidiploid by natural selection. Genotyping-by-sequencing (GBS) coverage analysis showed that improved fertility is associated with the elimination of entire and partial chromosomes carrying factors that either cause the disruption of plant development in hybrid plants or lead to the restoration of the euploid number of chromosomes (2n = 56) in the absence of one of the incompatible alleles. Highly fertile offspring obtained in compatible and incompatible crosses can be successfully adapted for the production of triticale pre-breeding stocks.

## Introduction

More than 130 years have passed since Rimpau^1^ obtained the first intergeneric hybrid combining the genomes of wheat and rye. This hybrid was genetically stable over 45 years^2^. By distant hybridization, the crop species triticale (× *Triticosecale* Wittmack) was created, and highly productive commercial varieties for various purposes were obtained^3^. Triticale is an alternative crop for areas characterized by low soil fertility as well as uneven and late rainfall^4^. However, the implementation of sustainable farming is impossible without obtaining new varieties of triticale that meet the requirements of modern farming^5,6^. To accomplish this goal, it is necessary to expand the genetic diversity of primary triticale. This can be achieved by introgressing favorable alleles, genes or gene complexes from wheat, rye and their wild relatives, which are responsible for qualitative traits, resistance to biotic and abiotic stresses, and heterosis^6–8^. However, not every combination of tetraploid or hexaploid wheat with rye results in a viable hybrid^2,9,10^. To overcome both prezygotic^11,12^ and postzygotic^13^ reproductive barriers between wheat and rye, different methods are being developed, including the transfer of recessive crossability alleles *kr1, kr2, skr* into well-adapted wheat varieties and in situ embryo rescue of abnormal embryos, yielding fertile amphidiploid plants. Furthermore, to optimize pre-breeding programs for triticale, it is necessary to develop effective methods for obtaining viable offspring^2,6,10^ and study the mechanisms of genome restructuring and ways to stabilize the new complex hybrid genome^14–17^.

Here, we monitor the process of genome stabilization of newly synthesized wheat-rye allopolyploids in early generations (R_0_, R_1_–R_3_) by using a bioinformatics approach based on sequence coverage along the genome derived from standard genotyping-by-sequencing (GBS) data as a proxy for genome integrity. We provide novel insights into the mechanism of allopolyploid formation, obtained in both compatible and incompatible crosses. The specific patterns of structural diversity observed within these types of crosses and the role of particular wheat and rye chromosomes in forming different traits in new allopolyploids are discussed.

## Material and methods

### Plant material and growth conditions

Intergeneric hybrids were generated between the hexaploid wheat (*Triticum aestivum* L., 2n = 6 ×  = 42) cultivar ‘Chinese Spring’ (CS) and two chromosomally modified lines of CS (nulli-tetrasomic line N6AT6D, referred to as line 31 and deletion line 6AL-8) as the female parents, and the self-fertile rye (*Secale cereale* L., 2n = 2 ×  = 14) line L2 from the unique Peterhof rye collection (St. Petersburg, Russia)^18,19^ as pollinator. L2 was obtained as a result of inbreeding (25 generations) of a line named ‘Monstrous Branched Ear’^20^ but having a normal ear phenotype (Fig. S1). It was widely involved in genetic analyses and mapping of self-fertility mutations in rye. In all intraspecific crosses with its participation, no postzygotic anomalies in the development of offspring generations were identified^20,21^. The wheat CS and nulli-tetrasomic line N6AT6D were kindly supplied by the John Innes Centre, Norwich (UK). The deletion line 6AL-8 of CS was kindly provided by Dr. W. Jon Raupp (Wheat Genetic & Genomic Resources Centre, Kansas State University, Manhattan, USA).

CS is a carrier of the *Eml-A1* allele located in the long arm of wheat chromosome 6A^22,23^, which interacts with the incompatible allele *Eml-R1b* of rye (line L2) and leads to embryo lethality. A cross between CS and the inbred rye line L2 is therefore classified as an incompatible cross. The amphihaploid wheat-rye plants from this cross were named AHL2, and amphidiploid plants as ADL2 (Fig. S1). Before flow cytometric analysis of the ploidy level, all plants from incompatible crosses, obtained after colchicine treatment, were numbered. In subsequent generations of fertile plants, only the number of GBS probes was used to label the offspring. The absence of the *Eml-A1* allele in CS lines N6AT6D (line 31 in our genetic stock) and 6AL-8 leads to the formation of viable hybrid grains; therefore, these intergeneric crosses with rye line L2 were classified as compatible. Amphihaploid plants from these crosses were named AH31L2 and AH6AL-8L2, and amphidiploid plants as AD31L2 and AD6AL-8L2, correspondingly. These plants were labeled with the number of GBS probe. From the incompatible cross (CS × L2), 229 amphidiploid plants (ADL2, R_0_) were obtained by in vitro embryo rescue, but only eight plants produced seeds^13^. The descendants of these eight ADL2 plants of R_0_, R_1_–R_3_ generations were cultivated under greenhouse and field conditions (Table 1). In addition, 44 AD31L2 (N6AT6DxL2) and 24 AD6AL-8L2 (6AL-8xL2) plants from compatible crosses were produced and analyzed. Amphidiploid plants from both compatible crosses were fertile, and the number of productive spikes and the number of grains per plant (GNP) were determined for each plant. Following traits of the main spike were studied: spike length (cm), number of spikelets, number of grains (GN), weight of grains in grams and seed set (%) in florets 1 and 2 of each developed spikelet. Reduced spikelets were not included in the fertility analysis. Thousand-grain weight (TGW) in grams was calculated based on the number of grains and the weight of the grains of the main spike. To reveal the range of somaclonal variability due to in vitro culture of immature embryos, regenerated plants of the maternal form CS were obtained, and the genotypes of seven CS (R_0_) randomly selected plants were analyzed by GBS.

**Table 1:** Pedigree of fertile primary amphidiploids obtained in incompatible cross of common wheat CS with inbred rye line L2.

Maternal plant R0	Generation								
Plant number	GNP (R0)	R0 (tc)^1)^				R1			
		Number tested plants	Location	Split: fertile-sterile	GNPMin-max	Numbertested plants	Location	Split: fertile-sterile	GNP min–max
93	20	5	IPK field	All fert.^2)^	1–17	6	St-P field	1:5	6
		**3(GBS)**	**IPK GH**	**2:1**	**0–25**	7	IPK field	All fert.	1–70
		–	–	–	–	**7(GBS)**	**IPK GH**	**6:1**	**0–54**
96	21	–	–	–	–	6	IPK field	5:1	0–10
						5	St-P field	All fert.	1–16
139	56	–	–	–	–	9	IPK field	8:1	1–61
						10	St-P field	8:2	1–29
		8	IPK field	All fert	2–93	**6(GBS)**	**IPK GH**	**All fert.**	**134–265**
						9	St-P field	All fert.	1–24
229	12	–	–	–	–	7	IPK field	6:1	5–32
233	2	–	–	–	–	1	IPK field	Fertile	13
250	3	–	–	–	–	**2(GBS)**	**IPK GH**	**All fert.**	**52–526**
245	0	**5(GBS)**	**IPK GH**	**All sterile**	**0.00**	–	–	–	–
264	9	–	–	–	–	8	IPK field	1:7	8
270	37	–	–	–	–	8	IPK field	7:1	0–13
						7	St-P field	5:2	0–18

Maternal plant R0	Generation								
Plant number	GNP (R0)	R2				R3			
		Number tested plants	Location	Split: fertile-sterile	GNP min–max	Number tested plants	Location	Split: fertile- sterile	GNP min–max
93	20	–	–	–	–	–	–	–	–
		–	–	–	–	–	–	–	–
		–	–	–	–	–	–	–	–
96	21	**7(GBS)**	**IPK GH**	**All fert.**	**14**–**139**	–	–	–	–
		–	–	–	–	–	–	–	–
139	56	–	–	–	–	–	–	–	–
		–	–	–	–	–	**–**	–	–
		–	–	–	–	**–**	–	–	–
		–	–	–	–	–	–	–	–
229	12	**5(GBS)**	**IPK GH**	**All fert.**	**51**–**209**	–	–	–	–
		8	St-P field	5:3	0–10	–	–	–	–
233	2	**5(GBS)**	**IPK GH**	**All fert.**	**1**–**88**	**10**	**IPK GH**	**7:3**	**0**–**146**
250	3	–	–	–	–	–	–	–	–
245	0	–	–	–	–	–	–	–	–
264	9	**8(GBS)**	**IPK GH**	**All fert.**	**2–301**	**GBS64**^3^ **11**	**IPK GH**	**3:8**	0–11
						**GBS65**^**3**^ **10**	**IPK GH**	**All fert.**	**97–810**
						**GBS67**^**3**^ **2**	**IPK GH**	**All sterile**	**0.00**
270	37	**5(GBS)**	**IPK GH**	**All fert.**	**25–263**	–	–	–	–
		–	–	–	–	–	–	–	–

(1) tc—regenerant plants obtained from immature inflorescences of maternal plant via tissue culture; (2) all tested plants were fertile; (3) Progeny R3 of individual plants (R2) selected based on GBS results; IPK field—the plants were grown under field conditions at Leibniz Institute of Plant Genetics and Crop Plant Research (IPK), Germany; IPK GH—the plants were grown under greenhouse conditions at Leibniz Institute of Plant Genetics and Crop Plant Research (IPK), Germany; St-P field—the plants were grown under field conditions at Sankt-Petersburg State University, the Russian Federation; GBS—the plants studied by genotyping-by-sequences are highlighted in bold. GNP—grains number per plant.

Plants were grown under greenhouse conditions at IPK (Gatersleben, Germany) in 2014, 2015, 2017 and 2018 and under field conditions in both Gatersleben and Sankt-Petersburg (Russia) in 2015. For greenhouse experiments, germinated seeds or plantlets of each cross were planted into 16 cm pots (one seed or plantlet per pot) and grown under 16/8 h day/night and temperature regime 20 °C/16 °C until harvest maturity stage following standard agronomic practices. For field experiments, 1–10 seeds of each 8 ADL2 plants of R_1_ and R_2_ generation (Table 1) were sown in 96-well trays and kept under greenhouse conditions (16/8 h day/night; 20 °C /16 °C) for 21 days. Starting at the tillering stage, plants were cultivated under field conditions, 10 plants per row of 100 cm with 30 cm between rows. Plants were manually irrigated, and standard agronomic practice was applied.

### In vitro embryo rescue, colchicine treatment and plant cloning procedure

To obtain hybrids, wheat spikes were emasculated 1–2 days before anthesis and pollinated 2–4 days later with fresh rye pollen. Hybrid embryos were excised 14 or 16 days after pollination (DAP), sterilized and cultivated, as reported by Tikhenko et al.^13^. Chromosome doubling was performed by in vitro colchicine application as described by Sood et al.^24^ with some modifications^13^. Two fertile plants (ADL2 plant 93 and ADL2 plant 139) and one sterile AD plant (ADL2 plant 245) from an incompatible cross were cloned using a culture of young inflorescences (Table 1). Additional tillers from fertile plants were surface sterilized with 70% ethanol for 1 min after which their outer leaves were removed^25^. Only inflorescences at the terminal spikelet stage^24^ or younger (4–20 mm long) were used for callus induction. Each inflorescence was cut into 2–5 explants, which were placed in 50 mm diameter Petri dishes. Embryogenic callus was obtained by culturing young inflorescence explants following the protocol for abnormal embryos^13^. Regenerated plantlets with developed roots were transplanted into potting soil and cultivated under greenhouse conditions.

### Flow cytometric analysis of the ploidy level

After the colchicine application in cell culture, the ploidy level of regenerated plants was determined twice, the first time before planting the plants in soil and the second time in the heading phase. Nuclei were isolated from roughly 0.5 cm^2^ leaf tissue either with a Polytron PT 1200 homogenizer (Kinematica AG, Littau, Switzerland) or by chopping with a sharp razor blade using the nuclei extraction and staining kit ‘CyStain UV ‘Ploidy’ (Code No. 05–5001; Sysmex-Partec, Görlitz, Germany) according to the manufacturer’s instruction. The resulting nuclei suspensions were filtered through a 50 µm CellTrics Disposable Filter (Code No. 04–0042-2317; Sysmex-Partec, Görlitz, Germany) and measured either on a Ploidy Analyzer PA flow cytometer (Partec GmbH, Münster, Germany) equipped with an Osram HBO 103 W/2 high-pressure mercury lamp or a FACStar^PLUS^ cell sorter (BD Biosciences, New Jersey, US) equipped with an Argon-ion laser (INNOVA 90C-5; Coherent, Santa Clara, US) adjusted to emit light in the UV range. For each sample, measurements were performed in two runs, measuring at least 2,000 nuclei per run. For each flow cytometry test, at least three amphihaploid plants were used, which were obtained without colchicine application and served as a reference standard for the determination of ploidy level.

### GBS analysis of parental forms, amphihaploid and amphidiploid plants

GBS libraries were prepared according to Wendler et al.^27^ and sequenced on an Illumina HiSeq2500 instrument at IPK Gatersleben. After adapter trimming with cutadapt, reads were mapped to the combined CS wheat reference genome version 1.0^28^ and the Lo7 rye reference genome version 1.0^29^ using BWA software package^30^. Alignment records were converted to BAM format with SAMtools^31^ and sorted according to alignment position with Novosort (http://www.novocraft.com/products/novosort/). The counts of uniquely mapped reads in non-overlapping 1 Mb bins of the CS and Lo7 references were determined with an AWK script and plotted along the genome in non-overlapping 5 Mb bins using plot functions of the R statistical environment (http://www.R-project.org/). For this, read counts were normalized in two steps. First, raw read counts in each genotype were divided by the sum of total read counts in that genotype to account for reading depth differences, and subsequently multiplied by 1e6. Second, log2-ratios between read counts in each bin between each sample and the average of the euploid CS x L2 controls were taken to account for coverage differences along the genome due to an uneven distribution of GBS targets. Accession numbers and mapping statistics of GBS samples are reported in Table S1.

## Results

### Structural chromosome variations in fertile wheat-rye hybrids from incompatible crosses

The genome composition of allopolyploids was analyzed using the GBS method. GBS analysis was carried out for 53 descendants (Tables 1, S2) of regenerated plants from incompatible crosses of generations R_0_ (designated plants 93 (p. 93) and 245 (p. 245)), R_1_ (plants 93, 139 and 250) and R_2_ (plants 96, 229, 233, 264 and 270). GBS coverage was analyzed to track structural changes associated with genome stabilization in these newly synthesized wheat-rye allopolyploids. Additionally, seven CS plants (R_0_) and 5 amphihaploid plants (AHL2 GBS 16–20) obtained from embryo culture without colchicine treatment (Table S2) were included for comparison, as well as five randomly selected maternal CS plants (GBS 1–5) and paternal plants of inbred rye line L2 (GBS 6–10) that served as internal controls (https://doi.ipk-gatersleben.de/DOI/b8b456bc-6f67-4380-89c7-5b01227a840c/40eab243-4421-4fba-bfc5-f7ad0148fd22/2/1847940088).

**Figure 1:**
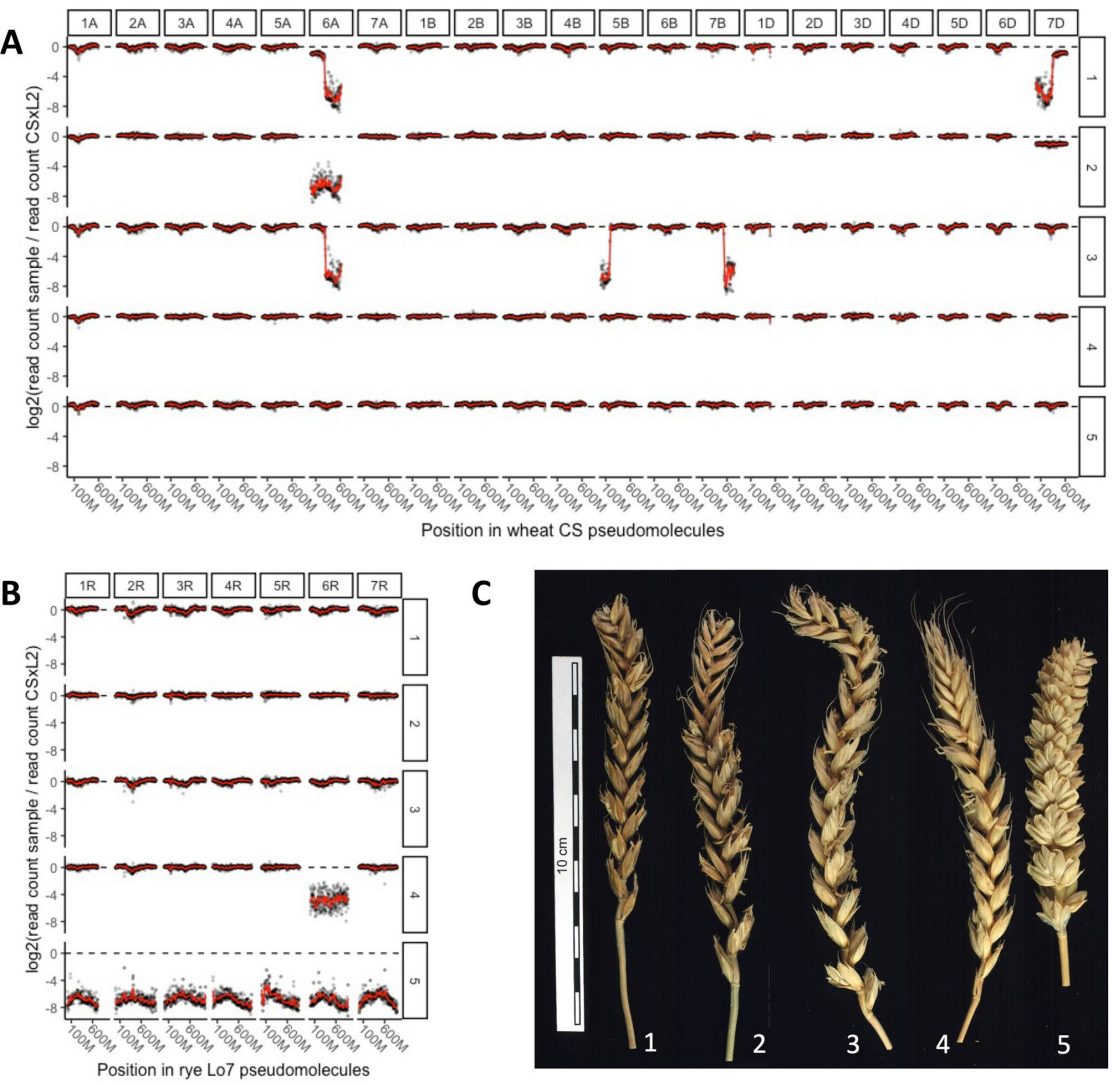
Normalized read coverage in 5 Mb bins along the wheat (**A**) and rye (**B**) genomes (CS V1.0 and Lo7 V1.0 reference assemblies, respectively), and spike morphology (**C**) for fertile intergeneric hybrids (ADL2) generated from incompatible crosses (CS × L2): (1) p. 93–23 GBS 32; (2) p. 93–18tc GBS 26; (3) p. 139/22–6 GBS 48; 4) p. 229/2–1 GBS 49; 5) p. 250/1–1 GBS 59. Negative log2 ratios indicate genome bins in which large scale deletions occurred in fertile intergeneric hybrids, including eliminations of the incompatible wheat allele *Eml-A1* on chromosome 6A and the incompatible rye allele *Eml-R1b* on chromosome 6R.

**Figure 2:**
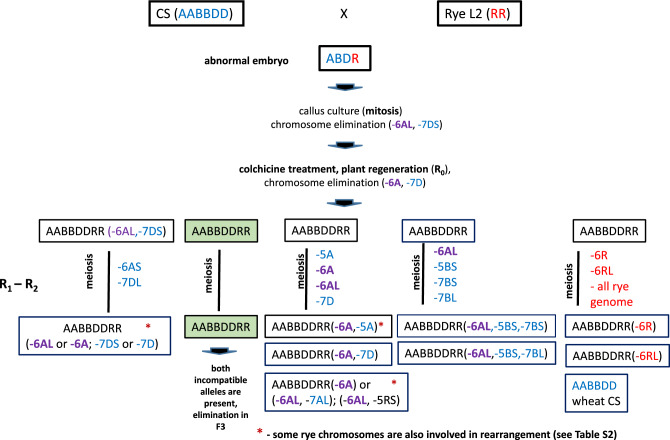
Overview of major chromosome rearrangements in intergeneric wheat-rye hybrids in generations R_0_, R_1_–R_2_ in incompatible crosses (CS × L2) detected by GBS coverage analysis.

The elimination of the incompatible wheat allele *Eml-A1* in offspring plants 93, 96, 139, 233, 250 and 270 or of rye allele *Eml-R1b* in plants 229 and 264 (Fig. 1, Table S2) is a prerequisite for the normal development of male and female gametophytes and seed formation in ADL2. Elimination of the incompatible allele can occur already in the somatic cells of the hybrid embryo (Fig. 2). In some somatic cells of in vitro cultured abnormal embryos, one arm of chromosome 6A or 6R on which the incompatible alleles *Eml-A1* or *Eml-R1b* are localized is missing. Somatic clones of plant 93 R_0_ (GBS 26–28, Table S2) were found to have lost the complete chromosome 6A (nullisomy) during cycles of mitotic divisions. In plants 233 (GBS 54–58), deletion of the terminal region of chromosome 6AL resulted in fertility.

In subsequent generations (R_1_ and R_2_), further loss of individual arms or whole chromosomes may occur during meiosis (Table 2, Fig. 2). These deletions usually involve wheat chromosomes (Tables S2, S4), which in *Triticum* species are actively involved in intragenomic and intergenomic translocations providing greater adaptability (Table S3). Likely, natural selection in subsequent generations occurred to increase the fertility of the amphidiploid. Enhanced fertility is associated with the elimination of chromosomes or chromosome regions bearing factors, whose presence disturbs the viability of hybrid plants. This is confirmed by the R_1_ descendants (GBS 43, 47 and 48) of plant 139, which form 238–265 GNP (grains per plant) and are double or triple ditelosomic for chromosomes 6A, 5B, and 7B (Table S2). The original plant R_0_ ADL2 139 had only 56 grains, and the 8 cloned plants of ADL2 p. 139 produced 2–93 GNP (Table 1).

**Table 2:** Spontaneous translocations in hexaploid wheat^1)^ and deletions of chromosomes or chromosome arms in intergeneric wheat-rye hybrids (R_0_–R_3_) in incompatible (ADL2) and compatible crosses (AD31L2; AD6AL-8L2) in R_0_ generations.

Ploidy level	Species or hybrids	Genome	Chromosomes involved in reconstruction						
6×	*T. aestivum* (AABBDD)^1)^	A		2A	3A	4A	5A		7A
8×	ADL2 (AABBDDRR)^2)^				3A	4A	5A	6A	7A
	AD31L2 (AABBDDRR)							**6A**^3)^	
	AD6AL-8L2 (AABBDDRR)							**6A**	**7A**
6×	*T. aestivum* (AABBDD)^1)^	B	1B	2B	3B	4B	5B	6B	7B
8×	ADL2 (AABBDDRR)^2)^		1B	2B		4B	5B	6B	7B
	AD31L2 (AABBDDRR)		1B			4B	5B		7B
	AD6AL-8L2 (AABBDDRR)		**1B**						
6×	*T. aestivum* (AABBDD)^1)^	D	1D	2D	3D	4D			7D
8×	ADL2 (AABBDDRR)^2)^			2D			5D		7D
	AD31L2 (AABBDDRR)			2D				**6D**	
	AD6AL-8L2 (AABBDDRR)					**4D**			
8×	ADL2 (AABBDDRR)^2)^	R	1R	2R	3R	4R	5R	6R	7R
	AD31L2 (AABBDDRR)		1R	2R			5R		
	AD6AL-8L2 (AABBDDRR)			2R		4R			

(1) Data are summarized based on the cited literature (see Table S3, which contains the cited literature); (2) 15 analyzed fertile plants R_2_ out of 48 had a set of chromosomes 8×  = 56, see Table S2; (3) Chromosome numbers in bold indicate the presence of changes in the number or structure of these chromosomes in mother plants, obtained as a result of direct selection.

Out of the 8 fertile plants from the incompatible cross CS × L2, six amphidiploid plants (plants 93, 96, 139, 233, 250, and 270) showed elimination of the incompatible wheat *Eml-A1* allele, while in two others (plants 229 and 264) the incompatible rye *Eml-R1b* allele was absent. In the R_2_ generation of the ADL2 plant 233, two plants (GBS 57 and 58) out of five lacked both incompatible allele. The absence of both incompatible alleles, however, may have a negative effect on fertility since these plants contained only 1–14 grains, while plants in which only the *Eml-A1* allele was eliminated (GBS 54–56) contained 18–88 grains (Table S2). Our comparative analysis of the DNA sequences of the descendants of the eight fertile plants from the incompatible cross (CS × L2) revealed a significant variation in the number of chromosomes. Only 15 plants displayed the complement of 56 chromosomes. Next to this, there were 7 plants with 55, 13 plants with 54, 7 plants with 53, 3 plants with 52 and 2 plants with 51 chromosomes, while one plant (GBS 59) only contained 42 chromosomes caused by the elimination of the complete rye genome (Table S2, Figs. 1 and 2). All fertile offspring with 56 chromosomes carried either a deletion(s) or loss of one of the arms of individual chromosomes; that is why they were mono-, di-, or tritelosomic (Table S2, Fig. 2). All five cloned descendants of the sterile plant ADL2 245 (GBS 21–25) had no major genome rearrangements (ADL2 p. 245, R_0_) and were completely sterile (Table S2, Fig. S1; https://doi.ipk-gatersleben.de/DOI/b8b456bc-6f67-4380-89c7-5b01227a840c/40eab243-4421-4fba-bfc5-f7ad0148fd22/2/1847940088).

To analyze the process of genome stabilization in the next generation (R_3_), we selected three ADL2 plants (plants 233/1-GBS 54, 264/3-GBS 64 and 264/6-GBS 67) which have the structure of 6A and 6R chromosomes statistically corresponding to the parental forms. The plant 264/4-GBS 65 was selected for being the most productive one (301 grains per plant) of all those analyzed in this incompatible cross.

Maternal plant ADL2 233/1-GBS 54 was monosomic for chromosome 4B and, in addition, carries deletions on the long arm of chromosomes 4AL and 6AL (Table S4, Fig. S2). The terminal deletion on 6AL suggests the loss of the incompatible allele *Eml-A1*, which allowed the plant to form 18 GNP. Seven out of ten offspring plants of ADL2 233/1-GBS 54 were ditelosomic, monosomic or nullisomic for chromosome 4B, which either caused a sharp decrease in the fertility (Dt4BS, M4B) or even sterility (N4B) in amphidiploid plants (GBS 153, 155–158, 161 and 162). Restoration of chromosome 4B disomy and the absence of the incompatible allele *Eml-A1* leads to the recovery of fertility (117–146 GNP) in two of the offspring plants (Figs. S2.2, S2.3; GBS 154 and 159). The loss of one copy of chromosome 3R in the GBS 154 plant did not strongly affect the plant’s productivity.

The maternal plant ADL2 264/3-GBS 64, possessing 53 chromosomes, was nullisomic for chromosome 1R and monosomic for chromosome 2R (Table S4). In addition, a deletion in the pericentric region of chromosome 1B was found. Since our GBS data indicate that chromosomes 6A and 6R are similar in structure to that of the parental forms (Fig. S3), this plant probably contains both incompatible alleles. Eight out of 11 offspring were nullisomic for chromosomes 1R and 2R. All had a strong spike reduction and were completely sterile (Fig. S3, GBS 163.). Three further offspring (GBS 166, 171, and 172) had one copy of chromosome 2R, like the maternal plant and formed 6–11 GNP (Table S4). GBS 172 showed monosomy for chromosome M3R.

Maternal plant ADL2 264/6-GBS 67 was monosomic for chromosomes 5D and 7R and carried a deletion in the pericentric region of chromosome 1B (Table S4, Fig. S4 GBS 67). We propose that the sterility of this plant is the result of the interaction of the incompatible alleles *Eml-A1* and *Eml-R1b*. The two offspring plants (GBS 174 and 175; Fig. S4) were sterile and had the same chromosome composition, except for an additional trisomy of chromosome 4B in GBS 174.

The euploid maternal plant ADL2 264/4-GBS 65 carried a deletion in the pericentric region of chromosome 1B (Table S4, Fig. S5 GBS 65). Four out of 10 offspring plants lost individual chromosomes of the D (1D, 5D) and/or the R (1R, 3R, 6R, 7R) genomes. Monosomy for chromosome 3R did not have negative consequences on plant’s productivity (GBS 176, Table S3). Monosomy of two chromosomes (5D and 1R or 6R and 7R) can be the reason for the decrease in grain productivity of offspring GBS 180 and 183 in comparison with the productivity of the maternal plant. Monosomy of chromosome 1D, which carries the *Dee-D1* locus, is critical for productivity. The loss of one copy of chromosome 1D reduced the TGW (shriveled seeds), caused fertility reduction (111 GNP) and a change in the spike morphology (Fig. S5, GBS 184). The remaining six descendants had a genome structure similar to that of the mother plant (https://doi.ipk-gatersleben.de/DOI/b8b456bc-6f67-4380-89c7-5b01227a840c/40eab243-4421-4fba-bfc5-f7ad0148fd22/2/1847940088). Five of them had higher productivity (370–810 GNP) compared to the mother plant. In comparison, only one plant (GBS185) showed a lower productivity (97 GNP) (Table S4).

### Structural chromosome variation and productivity of wheat-rye hybrids from compatible crosses

Two chromosomally modified lines of CS were used as maternal parents in our experiment. Modifications of their karyotypes were also studied by GBS. DNA fingerprinting profiles of both the nulli-tetrasomic line N6AT6D (Fig. S6, GBS 199) and the deletion line CS 6AL-8 (Fig. S8, GBS 200) showed significant differences from the CS profile (Fig. S1). Both genetically modified wheat lines were heterogeneous in the presence of additional deletions. Some maternal plants of the N6A/T6D line carried additional deletions in the short arm of chromosome 6D, and some had an additional deletion in the long arm of chromosome 7D. The deletion line 6AL-8, in addition to the expected deletion in the terminal region of the 6AL chromosome, carried additional deletions in chromosomes 7A, 1B and 4D (Table S6; https://doi.ipk-gatersleben.de/DOI/b8b456bc-6f67-4380-89c7-5b01227a840c/40eab243-4421-4fba-bfc5-f7ad0148fd22/2/1847940088). To analyze the structure of the amphihaploid genomes in crosses with the nulli-tetrasomic line, leaves of the plants generated from hybrid seeds were used for DNA isolation. For the CS 6AL-8 deletion line, the GBS profiles of haploid plants were obtained from plants regenerated via callus culture. In the case of amphihaploid plants obtained via the nulli-tetrasomic line, pattern alignment resembled those of the maternal and paternal forms (Fig. S6 GBS 208). Analysis of amphihaploid and amphidiploid plants from the cross CS 6AL-8 × L2 showed the presence of polymorphism in the number of additional deletions in the genotype of the tested line. This line contains plants with three and four deletions (Fig. S8). One randomly selected plant of CS 6AL-8 with four deletions was sequenced as a standard. GBS revealed that in each cross, the plants from groups with different spike fertility shared similar but not identical profiles.

Forty-four amphidiploid plants (AD31L2) were obtained from the compatible cross CS N6AT6D × L2 after embryo culture (Table S5). The maternal line was heterogeneous in the number of deletions in chromosomes 6D and 7D. Five amphihaploid plants, obtained directly from hybrid seeds, showed three different haplotypes (https://doi.ipk-gatersleben.de/DOI/b8b456bc-6f67-4380-89c7-5b01227a840c/40eab243-4421-4fba-bfc5-f7ad0148fd22/2/1847940088). The maternal plant of GBS 208 was nullisomic for chromosome 6A and tetrasomic for chromosome 6D (Fig. S6.1). Maternal plants of GBS 206, 207, and 210 were nullisomic for 6A and tetrasomic for 6D with a deletion on the short arm of all four copies of this chromosome. The maternal plant of GBS 209 was nulli-tetrasomic N6AT6D with deletions on the short arm of chromosome 6D and on the long arm of chromosome 7D (Table S5). Only the first and second haplotypes of the maternal line (Table S6) present in amphidiploids were obtained in this cross. In this compatible cross, 41 amphidiploid plants out of 44 were euploid (2n = 56 chromosomes), two plants were found to have 55, while one plant had 54 chromosomes (Table S5).

Forty-three plants out of 44 amphidiploids (R_0_) were fertile. In 22 of these, the main spike had a fertility rate above 50%, producing 162.5–193.7 GNP (Table S7, Fig. S6 GBS 236, GBS 254). The presence of a deletion on the short arm of 6D (haplotype 2) led to a significant decrease in fertility (101.3 GNP) compared to haplotype 1 (162.5–286.1 GNP, Tables S7, S9). The main spike of plants belonging to haplotype 1, having no chromosomal changes, had the highest fertility rate of the main spike (> 70%) (Tables S5, S7). The GBS 219 plant, carrying only the *Eml-R1b* allele of rye, confirmed our assumption that the sterility of wheat-rye hybrids in the cross CS with L2 is the result of the presence of incompatibility alleles (*Eml-A1* and *Eml-R1b*) of both parents. This R_0_ plant (Fig. S7, GBS 219) had a stem height of 20 cm and an ear with four spikelets carrying 2 grains. The two offspring of this plant (GBS 263 and 264) formed 204 and 186 GNP, respectively (Fig. S7, GBS 263 and 254). Significant changes in the structure of the rye genome were noted only in plants GBS 258 and GBS 264 (Table S5, https://doi.ipk-gatersleben.de/DOI/b8b456bc-6f67-4380-89c7-5b01227a840c/40eab243-4421-4fba-bfc5-f7ad0148fd22/2/1847940088). They can be classified as terminal deletions on chromosomes 1R and 5R in the plant GBS 258 and a loss of the short arm of both chromosomes 1R and monosomy of chromosome 2R in plant GBS 264. In seven plants with the fertility of the main spike below 49%, we noted either the single loss of the long arm in one of the chromosomes of the B genome: 1B, 4B, 5B, 7B or monosomy for chromosome 1B (Table S5).

In crosses between the CS deletion line 6AL-8 with inbred rye line L2, 25 amphidiploids (AD6AL-8L2) were produced (Tables S8, S10). All AD6AL-8L2 (R_0_ generation) plants carried 56 chromosomes. The heterogeneity of the maternal line CS 6AL-8, containing numerous deletions in chromosomes of the wheat genome next to the emergence of new deletions on the short arm of chromosome 2RS and the long arm of 4RL, led to an increase in the diversity of amphidiploid haplotypes. In total 5 major haplotypes were identified in AD6AL-8L2 (Table S6). Most of the amphidiploid (23 plants) were obtained from crossing maternal wheat plants carrying deletions on chromosomes 6AL, 1BL, and 4DS, and two plants (GBS 275 and GBS 289) from crossing with CS 6AL-8 plants carrying an additional deletion on chromosome 7AL (Fig. S8, GBS 200). The seven amphidiploid plants out of 25 with four different haplotypes showed fertility of the main spike. More than 50% formed on average 347.0–403.7 GNP (Tables S7, S10). All additional or multiple indels in the chromosome structure of wheat and rye chromosomes in amphidiploids from both compatible crosses (excluding plants GBS 263 and GBS 264) and amphihaploids (excluding plants GBS 206–210) are caused by mitotic chromosomal rearrangements during callus formation and subsequent somatic embryo- and organogenesis.

## Discussion

Compared with many other allopolyploids, octoploid triticale is more complex because of its high ploidy level, large genome size and the distant relationship between wheat and rye^14^. To understand early evolutionary events, we analyzed newly synthesized wheat-rye allopolyploids to investigate early genetic changes contributing to the genome stabilization process of allopolyploids from compatible (N6AT6DxL2, 6AL-8 × L2) and incompatible (CS × L2) crosses.

GBS coverage analysis represents a convenient and fast method for analyzing the genome composition of allopolyploids^32,33^. We found that the presence of parental incompatible alleles triggers major rearrangements in wheat and rye chromosomes in wheat-rye hybrids. Three general types of structural chromosome rearrangements in fertile ADL2 plants and their offspring were identified by GBS. They included: (1) chromosomal deletions (terminal, interstitial or pericentric), (2) elimination of one chromosome arm and (3) loss of one chromosome or both homologous chromosomes. Genome restructuring could result in the elimination of either the wheat (*Eml-A1*) or rye (*Eml-R1b*) incompatible alleles (Fig. 1). In 3.5% of cases (8 plants out of 229), structural changes were observed in the karyotype of somatic cells belonging to the hybrid embryo. These changes included a deletion of the subtelomeric region of the long arm of chromosome 6A and the elimination of the long arm of 6A or the long arm of 6R (Table S2). Progeny of such cells forms organogenic centers or somatic embryos in the callus culture, giving rise to regenerated plants capable of forming viable seeds. During the formation of adventive buds of additional tillers, the loss of the second arm of this chromosome may also occur, leading to nullisomy for this chromosome in plants GBS 26–28 (Table S2). As was shown earlier, allopolyploidization is accompanied by rapid inter/intra-genomic exchange^15^, which may have occurred in somatic cells^34^. Unequal chromosome divisions lead to chromosome elimination during mitosis. The fast stability of allopolyploids was confirmed in newly synthesized wheat-rye hybrids^35^. In our experiments during embryo culture, simultaneously with the process of the elimination of chromosomes carrying incompatible alleles, restructuring of parental genomes takes place due to duplication (trisomy of chromosome 4B) or elimination of arms or individual chromosomes of wheat and rye (GBS 26–28, Table S2). The process of genome reconstruction is activated during sexual reproduction, and in subsequent generations (R_1_–R_3_) during meiosis (Fig. 2) and more and more favorable rearrangements appear that provide the descendants with higher fertility (Tables 2, S2, S4). Thus, increasing fertility is associated with the eliminations of entire chromosomes or of regions bearing factors detrimental to plant development (descendants GBS 43, 47 and 48) or restoration of the euploid number of chromosome (plants GBS 154 and 159). Probably, changes in the structure of wheat chromosomes 5B and 7B in plants GBS 43, 47 and 48 are one of the mechanisms to stabilize the intergenomic relationships in newly formed wheat-rye allopolyploids. Numerous changes in the structure of some wheat chromosomes, including 5B and 7B were found in the hexaploid triticale N9116H and N9116M, which were selected for high productivity^16^. Similar structural changes in the wheat chromosomes 5A, 6A, 1B, 2B, 6B, 7B, 1D, 3D and 7D were observed in the progeny from crossing octoploid triticale lines with common wheat and subsequent self-pollination of these hybrids^35^. Our results confirm the previously forwarded assumption by Badaeva et al.^36^ and Feldman et al.^37^, that asymmetric deletions or elimination of individual regions or entire chromosomes in ADL2 plants in R_0_–R_3_ generations act as a unique mechanism of adaptation and stabilization of genomes in newly synthesized wheat-rye allopolyploids. The relationship between the parental genomes is the key factor in determining the direction, amount, timing, and rate of genomic sequence variation occurring during intergeneric allopolyploidization.

Hybrid lethality and sterility arising in the cross CS × L2 can be overcome using the wheat nulli-tetrasomic N6AT6D line and the deletion 6AL-8 line. Allopolyploids AD31L2 and AD6AL-8L2 are fertile plants. As shown earlier, in primary octoploid triticale of the early generations (C_1_–C_3_), seed setting is lower (0.1–40%) than that in hexaploid triticale (20–65%)^38,39^. However, even in euploid plants of the late generations (C4–C5) of primary hexaploid triticale, this parameter ranged from 23 to 76% (59% on average)^40^. In both compatible crosses performed here, the seed set in the main spike of primary octoploid triticales was more than 50%. The seed set in the main spike of amphidiploid plants was 72.7–82.2%, while seed production for AD31L2 ranged between 162.5–193.7 grains per plant and that for AD6AL-8L2 between 347 and 403.7 grains. The higher number of GNP of AD6AL-8L2 plants in comparison with AD31L2 plants is caused by their greater tillering capacity (Table S7).

Both maternal wheat lines are not homogeneous, and the derived amphihaploids and amphidiploids are represented by several haplotypes (Table S6). It can be assumed that genome imbalance resulting from the replacement or loss of wheat chromosome 6A or its subtelomeric region results in additional chromosome changes in maternal plants. Additional genome rearrangements in wheat-rye hybrids derived from these lines are the result of structural changes in the process of somatic cell division (Tables S5, S8). In both compatible crosses, half of the offspring showed a decrease in fertility (Table S7). A preliminary comparative analysis of the sequences (not shown here) indicates that the decrease in productivity of the amphidiploid R_0_ plants from compatible crosses is a consequence of the accumulation of numerous small chromosome rearrangements (indels) in both wheat and rye genomes (https://doi.ipk-gatersleben.de/DOI/b8b456bc-6f67-4380-89c7-5b01227a840c/40eab243-4421-4fba-bfc5-f7ad0148fd22/2/1847940088). There is no doubt that the study of the inheritance of grain productivity and possible processes of genome reorganization in the descendants of R_2_–R_3_ of these compatible crosses will provide new information on the coevolution of wheat and rye genomes in triticale. Highly fertile offspring obtained in compatible crosses can be successfully adapted for the production of the triticale pre-breeding stocks.

The genetic and epigenetic mechanisms of gene expression and phenotypic variability in plant polyploids are actively studied^41,42^. Hexaploid wheat, as most allopolyploid plants, has evolved over thousands of years, experienced multiple rounds of hybridization and genome duplication events, and has an ambiguous origin^43–45^. Newly synthesized hybrids with known parents are excellent material for dissecting gene expression and genomic changes in the early stages of allopolyploidization^46–48^. Furthermore, colchicine treatment, widely used in polyploid induction, tissue culture and plant regeneration, can introduce mutation and epigenetic changes^49^. These changes might influence gene expression and eventually interfere with the accuracy of studies on ploidy effects^42^. Environmental conditions can also determine the nature of gene expression in recent allopolyploids. The relative expression of homeologous genes varied depending on the gene, the organ, and the growing condition^50,51^. Taking into account the possible influence of all these factors, we were able to characterize the role of certain wheat and rye chromosomes in the formation of individual traits in newly synthesized ADL2 allopolyploids.

The important role of wheat chromosome 4B in productivity should be noted, although we did not reveal major changes in its structure. Monosomy on chromosome 4B leads to a significant decrease in GNP in allopolyploids, and its complete absence (nullisomy) can cause plant sterility (p. GBS 30–31, 54, 58, Table S2). The only completely sterile plant in the N6AT6D × L2 compatible cross (AD31L2 p. GBS 250) was monosomic for chromosome 4B. At the same time, the restoration of disomy of chromosome 4B and the absence of the incompatible allele *Eml-A1* leads to the restoration of fertility (117–146 GNP) in offspring (GBS 154 and 159) of the aneuploid plant ADL2 p. 233/1 GBS 54. This suggests an important regulatory function of this chromosome in the new complex genome.

Recently, we identified and mapped a novel *DEFECTIVE ENDOSPERM–D1* (*Dee-D1*) locus on chromosome arm 1DL that is involved in the genetic control of endosperm development^52^. The absence of *Dee-D1* leads to non-viable grains in distant crosses and alters grain shape, which negatively affects GN and TGW. *Dee-D1* can be classified as a speciation locus with a positive effect on the function of genes, which are involved in endosperm development in hybrid genomes. The presence of two copies of *Dee-D1* is necessary for normal endosperm development and thus plays an essential role in the evolution and improvement of grain yield in hexaploid wheat. Monosomy of chromosome 1D, which carries the *Dee-D1* locus, is critical for plant productivity in octoploid triticale (GBS 184). The loss of this chromosome reduces the TGW by 20% and the fertility of the spike by up to 15% associated with a change in the morphology of the spike (Table S4; Fig. S5.4). This study confirms the necessity of the presence of two doses of this locus for the normal development of the endosperm in primary octoploid triticale.

We noted the most significant phenotypic effect in the descendants of the GBS 64 plant of the R_3_ generation, in which there was a complete loss of chromosome 2R. Nullisomy on chromosome 2R leads to a strong reduction in spikelet number per spike and sterility in the offspring of the plant ADL2 p. 264/3 GBS 64 (Table S4, Fig. S3.2). Cheng and Murata^51^ have shown that selection for agronomic traits leads to the loss of chromosomes 2R and 5RS in octoploid triticale. The loss of rye chromosomes was compensated by an extra pair of 2D or A-genome chromosomes (possibly 2A) in plants with 2n = 56. The authors suggested that the loss of 2R and 5RS with their replacement by chromosomes of the D and A genome contributes to the improvement of octoploid triticale^33,53^. The vast changes in spike architecture caused by nullisomy of the 2R and 5R chromosomes of rye were found in androgenic offspring of hexaploid triticale (× *Triticosecale* Wittmack). Nullisomy of chromosomes 2R and 5R may correlate with the location of the major QTLs for spike morphology traits on these chromosomes^54^.

An important role of chromosome 6R in the triticale genotype was first shown in the studies by Merker^55^. Most of the 50 advanced lines with good agronomic characters had a mixed chromosome composition, but chromosome 6R was present in all tested triticale lines. In our case, 6R is one of the main chromosomes in determining the fate and viability of wheat-rye hybrids. The interaction between incompatible alleles of hexaploid wheat *Eml-A1* and rye *Eml-R1b* leads to the formation of reproductive barriers at different stages of development (embryo lethality and hybrid sterility) in wheat-rye hybrids. The loss of both *Eml* loci leads to a sharp decrease in plant productivity (plants GBS 57 and 58, Table S2). The integration of an EST-derived rye SSR marker enabled comparative mapping, and revealed that *Eml-R1* is located on an interstitial region on chromosome 6RL, covering a known 3L/6L translocation breakpoint^56^. The authors suggested that the *Eml* genes may have orthologs on chromosomes of the homeologous group 6 in different species of the tribe Triticeae, including species with a sequenced genome. They assumed that the genes of developmental pathways could be classified as a separate class of plant genes that can serve as causal genes of reproductive isolation^56^. Thus, the possibility of obtaining wheat-rye hybrids in incompatible crosses opens up broad prospects for understanding the mechanisms of reproductive isolation and evolution of both rye and wheat karyotypes and genomes.

### Statement

We confirm that experimental research and field studies on plants (either cultivated or wild), including the collection of plant material, comply with relevant institutional, national, and international guidelines and legislation.

### Supplementary Information

Supplementary Information 1.

Supplementary Information 2.

Supplementary Information 3.

Supplementary Information 4.

Supplementary Information 5.

Supplementary Information 6.

Supplementary Information 7.

Supplementary Information 8.

Supplementary Information 9.

Supplementary Information 10.

Supplementary Information 11.

Supplementary Information 12.

Supplementary Information 13.

Supplementary Information 14.

Supplementary Information 15.

Supplementary Information 16.

Supplementary Information 17.

Supplementary Information 18.

## Data Availability

Genotyping-by-sequencing raw data were deposited in the European Nucleotide Archive (accession: PRJEB64645). Read depth statistics are accessible under DOI http://dx.doi.org/10.5447/ipk/2024/4.
