# Supplementary material for: Major chromosome rearrangements in intergeneric wheat × rye hybrids in compatible and incompatible crosses detected by GBS read coverage analysis
Source: Sci Rep. 2024 May 14;14:11010. doi: 10.1038/s41598-024-61622-1 (PMC11094192; doi:10.1038/s41598-024-61622-1)
Supplement: Supplementary file 1 — Supplementary Information 1. [file 41598_2024_61622_MOESM1_ESM.pdf]

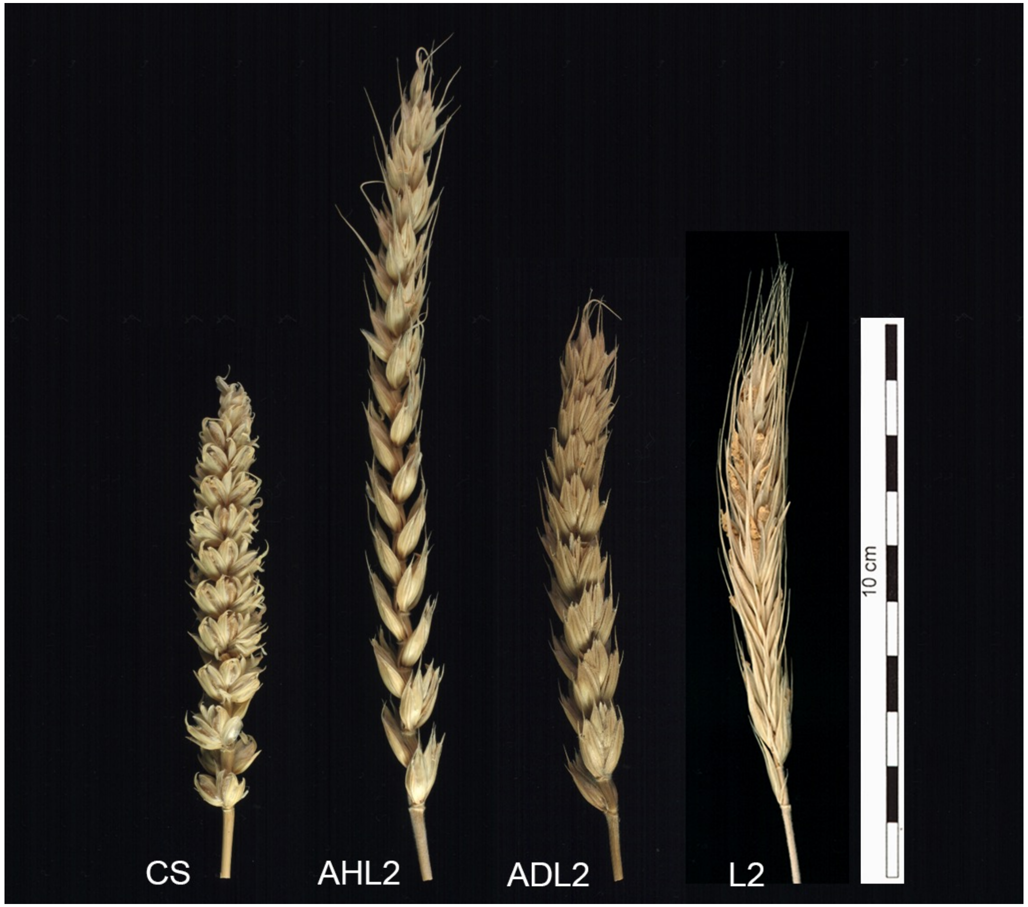

Fig. S1 The spike phenotypes:
1. CS - maternal plant Chinese Spring;
2. AHL2 - amphihaploid from cross CS x L2, sterile;
3. ADL2 - sterile amphidiploid p.245 from cross CS x L2;
4. L2 - paternal rye inbred line L2.

Normalized read coverage in 5 Mb bins along the wheat and rye genomes (Chinese Spring V1.0 and Lo7 V1.0 assembly)

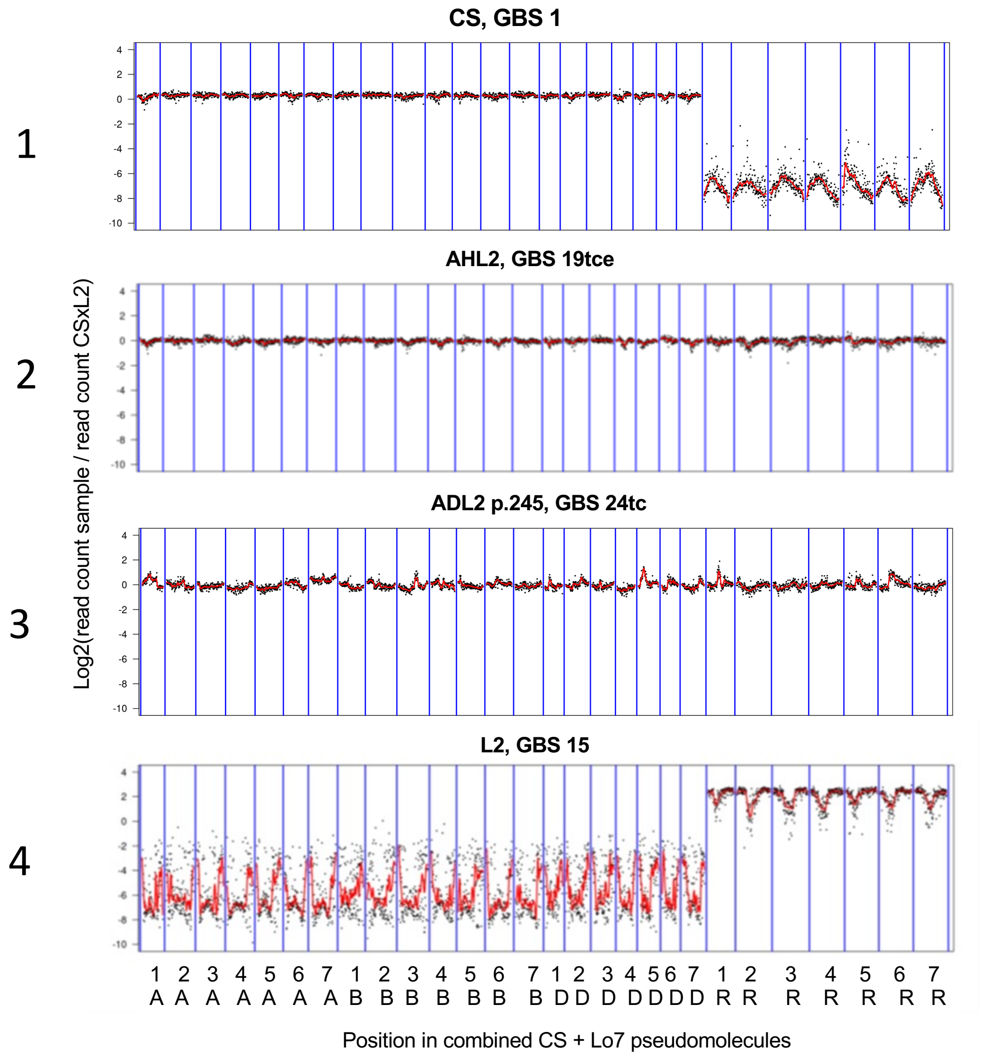

Fig. S1: Spike morphology and normalized read coverage in 5 Mb bins along the wheat and rye genomes (CS V1.0 and Lo7 V1.0 reference assemblies, respectively) for parental forms and intergeneric hybrids: 1) CS GBS 1, 2) AHL2 GBS 19tce, 3) ADL2 p.245/4 GBS 24tc, 4) L2 GBS 15.
