# Supplementary material for: Major chromosome rearrangements in intergeneric wheat × rye hybrids in compatible and incompatible crosses detected by GBS read coverage analysis
Source: Sci Rep. 2024 May 14;14:11010. doi: 10.1038/s41598-024-61622-1 (PMC11094192; doi:10.1038/s41598-024-61622-1)
Supplement: Supplementary file 2 — Supplementary Information 2. [file 41598_2024_61622_MOESM2_ESM.pdf]

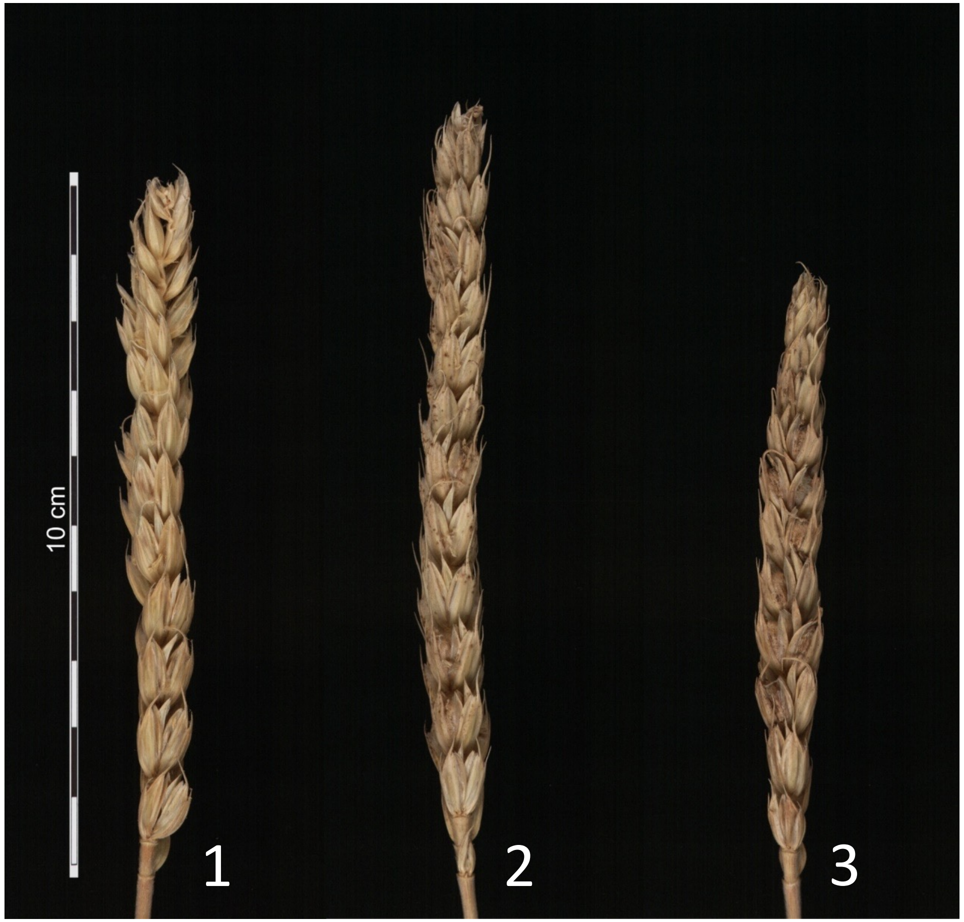

Normalized read coverage in 5 Mb bins along the wheat and rye genomes (Chinese Spring V1.0 and Lo7 V1.0 assembly)

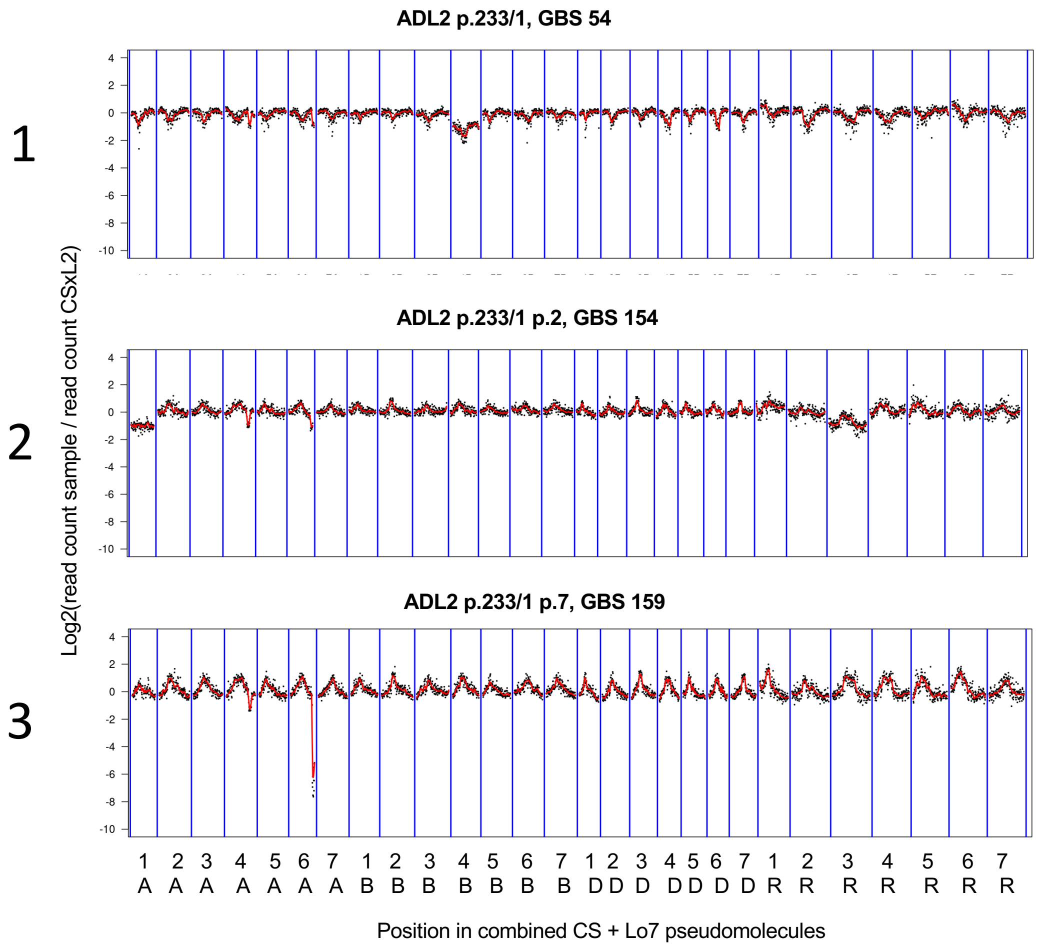

Fig. S2 The spike phenotypes of the fertile plant ADL2 p.233/1 GBS 54 and its descendants from self-pollination:

1. - ADL2 p.233/1 GBS 54
2. - ADL2 p.233/1 p.2 GBS 154
3. - ADL2 p.233/1 p.7 GBS 159

Fig. S2: Spike morphology and normalized read coverage in 5 Mb bins along the wheat and rye genomes (CS V1.0 and Lo7 V1.0 reference assemblies, respectively) of the fertile plant ADL2 p.233/1 GBS 54 and its descendants from self-pollination: 1) ADL2 p.233/1 GBS 54, 2) ADL2 p.233/1 p.2 GBS 154, 3) ADL2 p.233/1 p.7 GBS 159.
