# Supplementary material for: Major chromosome rearrangements in intergeneric wheat × rye hybrids in compatible and incompatible crosses detected by GBS read coverage analysis
Source: Sci Rep. 2024 May 14;14:11010. doi: 10.1038/s41598-024-61622-1 (PMC11094192; doi:10.1038/s41598-024-61622-1)
Supplement: Supplementary file 4 — Supplementary Information 4. [file 41598_2024_61622_MOESM4_ESM.pdf]

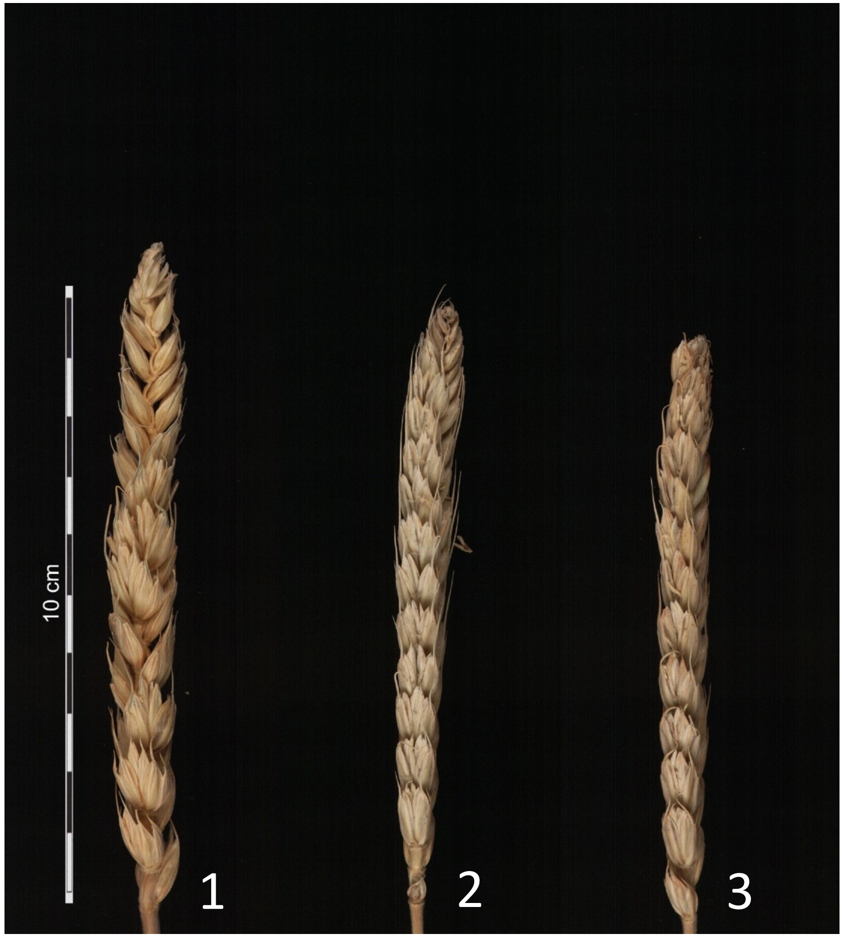

Fig. S4 The spike phenotypes of the fertile plant ADL2 p.264 / 6 GBS 67 and its descendants from self-pollination:

1. ADL2 p.264/6 GBS 67
2. ADL2 p.264/6 p.1 GBS 174
3. ADL2 p.264/6 p.2 GBS 175

Normalized read coverage in 5 Mb bins along the wheat and rye genomes (Chinese Spring V1.0 and Lo7 V1.0 assembly)

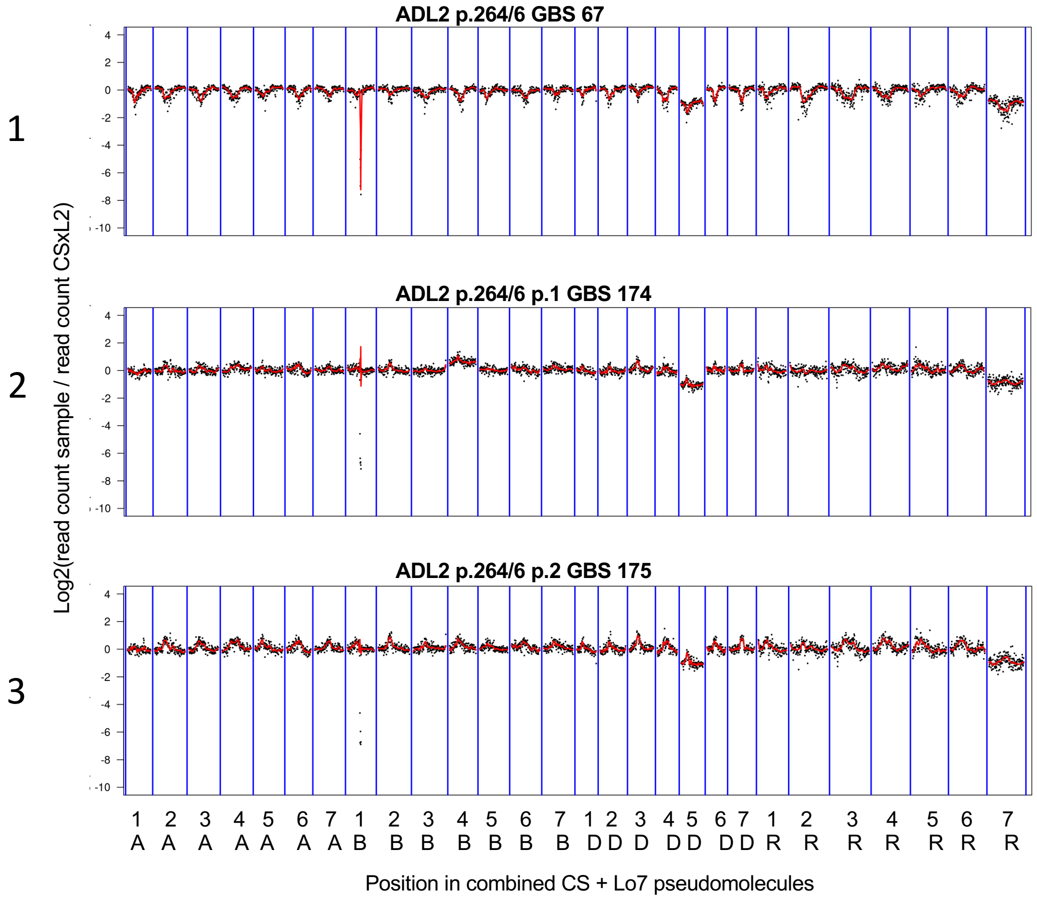

Fig. S4: Spike morphology and normalized read coverage in 5 Mb bins along the wheat and rye genomes (CS V1.0 and Lo7 V1.0 reference assemblies, respectively) of the fertile plant ADL2 p.264/6 GBS 67 and its descendants from self-pollination: 1) ADL2 p.264/6 GBS 67, 2) ADL2 p.264/6 p.1 GBS 174, 3) ADL2 p.264/6 p.2 GBS 175.
