# Supplementary material for: Major chromosome rearrangements in intergeneric wheat × rye hybrids in compatible and incompatible crosses detected by GBS read coverage analysis
Source: Sci Rep. 2024 May 14;14:11010. doi: 10.1038/s41598-024-61622-1 (PMC11094192; doi:10.1038/s41598-024-61622-1)
Supplement: Supplementary file 6 — Supplementary Information 6. [file 41598_2024_61622_MOESM6_ESM.pdf]

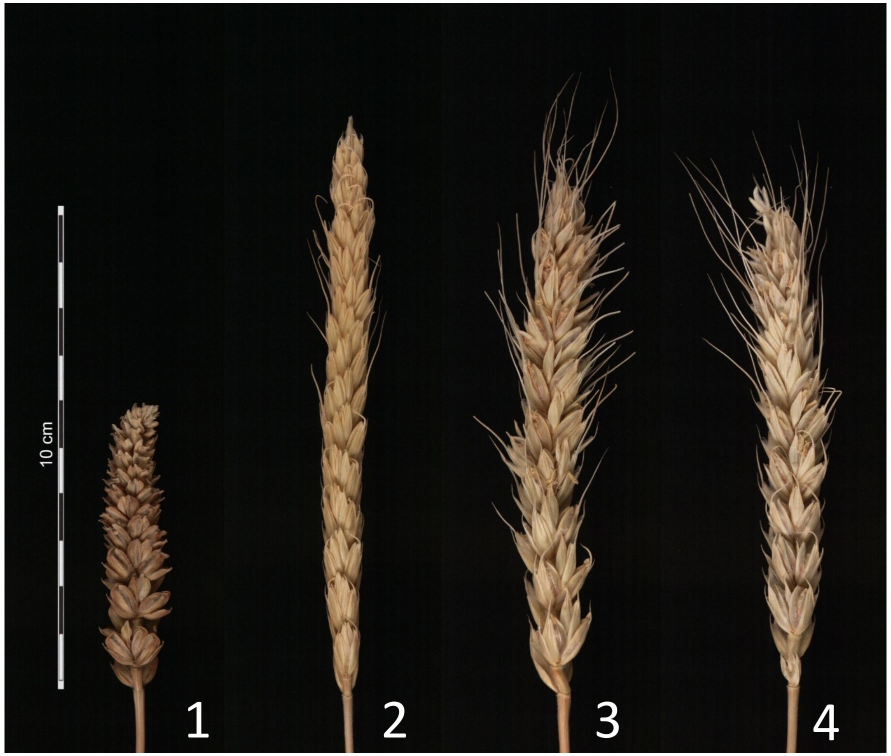

Fig. S6 The spike phenotypes of maternal wheat plant N6AT6D, amphihaploid and amphidiploid from cross with rye inbred line L2 with *Eml-R1b* allele:

1. wheat nulli-tetrasomic N6AT6D line GBS 199 (2n=42)
2. AH31L2 p.3 GBS 208 (n=28)
3. AD31L2 p.26/4 GBS 236 (2n=56)
4. AD31L2 p.46/1 GBS 254 (2n=56)

Normalized read coverage in 5 Mb bins along the wheat and rye genomes (Chinese Spring V1.0 and Lo7 V1.0 assembly)

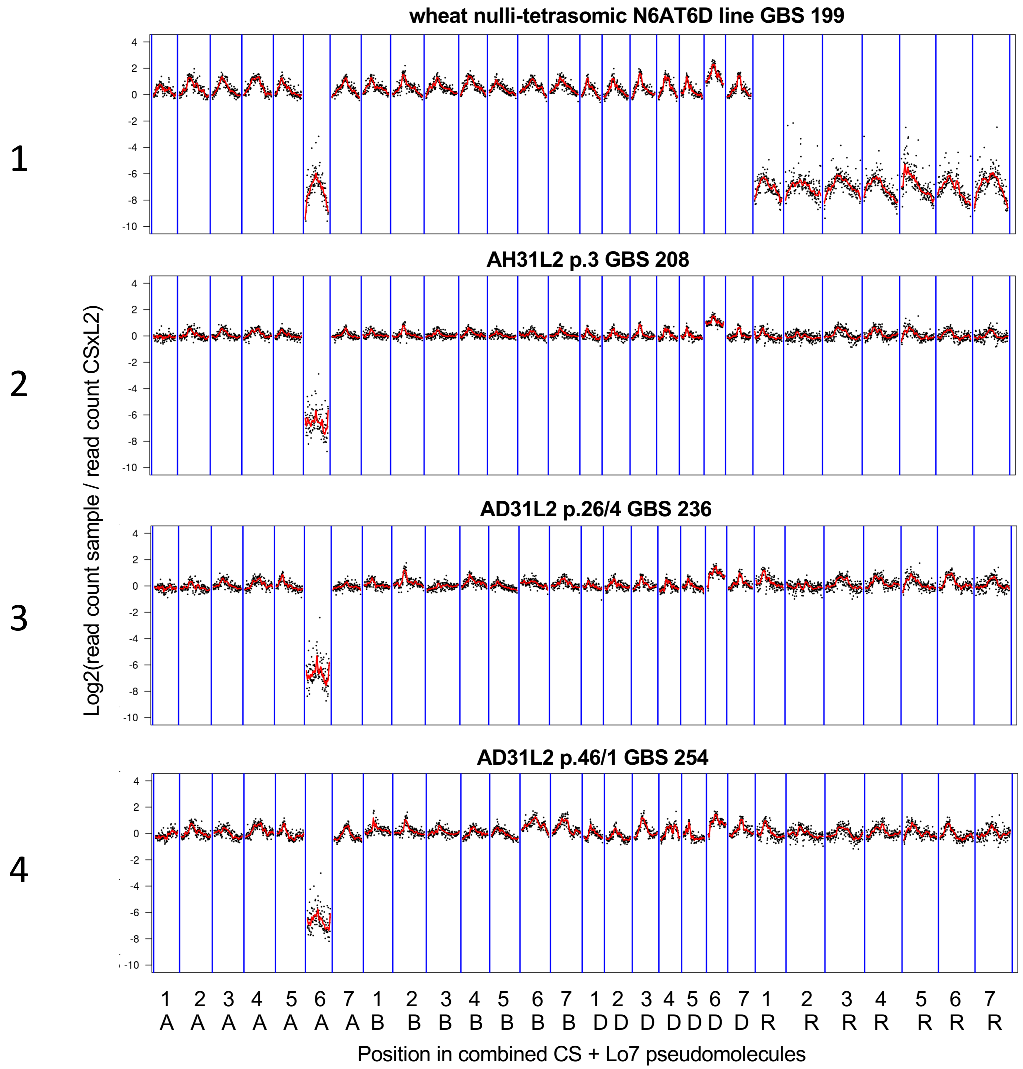

Supplementary Fig. S6: Spike morphology normalized read coverage in 5 Mb bins along the wheat and rye genomes (CS V1.0 and Lo7 V1.0 reference assemblies, respectively) of maternal wheat plant N6AT6D, amphihaploid and amphidiploids from cross N6AT6D x L2: 1) wheat nulli-tetrasomic N6AT6D line GBS 199, 2) AH31L2 p.3 GBS 208s, 3) AD31L2 p.26/4 GBS 236, 4) AD31L2 p.46/1 GBS 254.
