# Supplementary material for: Major chromosome rearrangements in intergeneric wheat × rye hybrids in compatible and incompatible crosses detected by GBS read coverage analysis
Source: Sci Rep. 2024 May 14;14:11010. doi: 10.1038/s41598-024-61622-1 (PMC11094192; doi:10.1038/s41598-024-61622-1)
Supplement: Supplementary file 7 — Supplementary Information 7. [file 41598_2024_61622_MOESM7_ESM.pdf]

Normalized read coverage in 5 Mb bins along the wheat and rye genomes (Chinese Spring V1.0 and Lo7 V1.0 assembly)

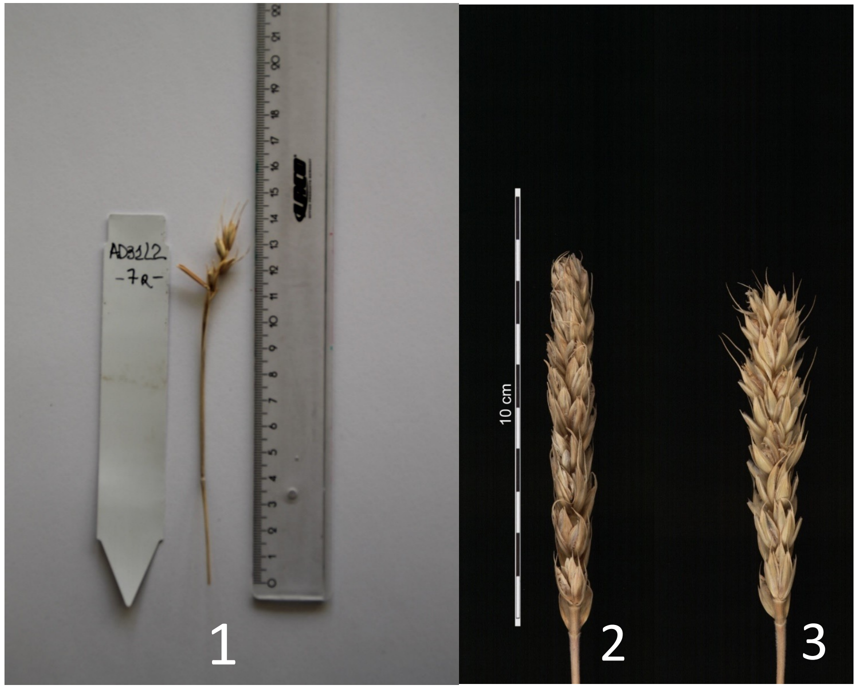

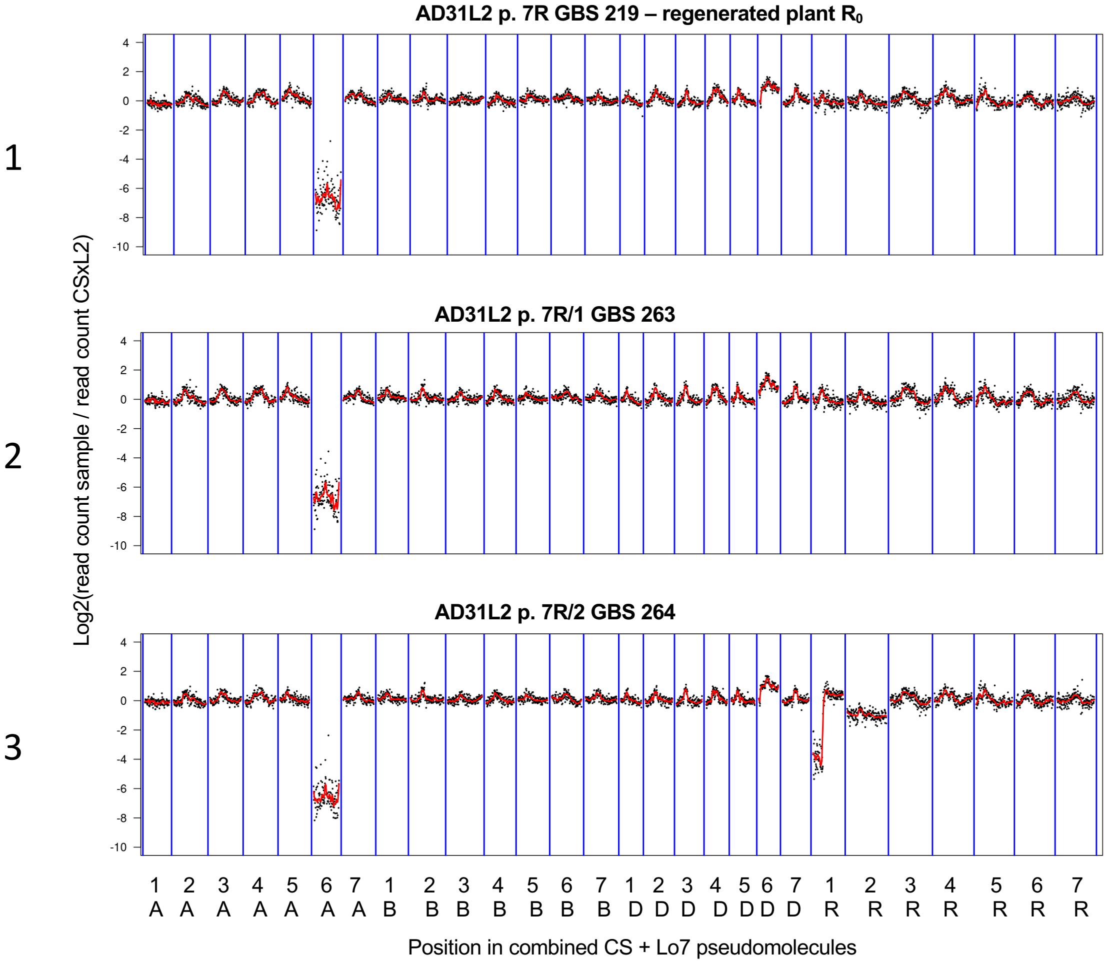

Fig. S7 The spike phenotypes of a fertile amphidiploid plant AD31L2 p.7R GBS 219 and its descendants from self-pollination:

1. AD31L2 p. 7R GBS 219 – regenerated plant R_0
2. AD31L2 p. 7R/1 GBS 263
3. AD31L2 p. 7R/2 GBS 264

Fig. S7: Spike morphology and normalized read coverage in 5 Mb bins along the wheat and rye genomes (CS V1.0 and Lo7 V1.0 reference assemblies, respectively) of a fertile amphidiploid plant AD31L2 p.7R GBS 219 and its descendants from self-pollination: 1) AD31L2 p. 7R GBS 219, 2) AD31L2 p. 7R/1 GBS 263, 3) AD31L2 p. 7R/2 GBS 264.
