# Supplementary material for: Major chromosome rearrangements in intergeneric wheat × rye hybrids in compatible and incompatible crosses detected by GBS read coverage analysis
Source: Sci Rep. 2024 May 14;14:11010. doi: 10.1038/s41598-024-61622-1 (PMC11094192; doi:10.1038/s41598-024-61622-1)
Supplement: Supplementary file 8 — Supplementary Information 8. [file 41598_2024_61622_MOESM8_ESM.pdf]

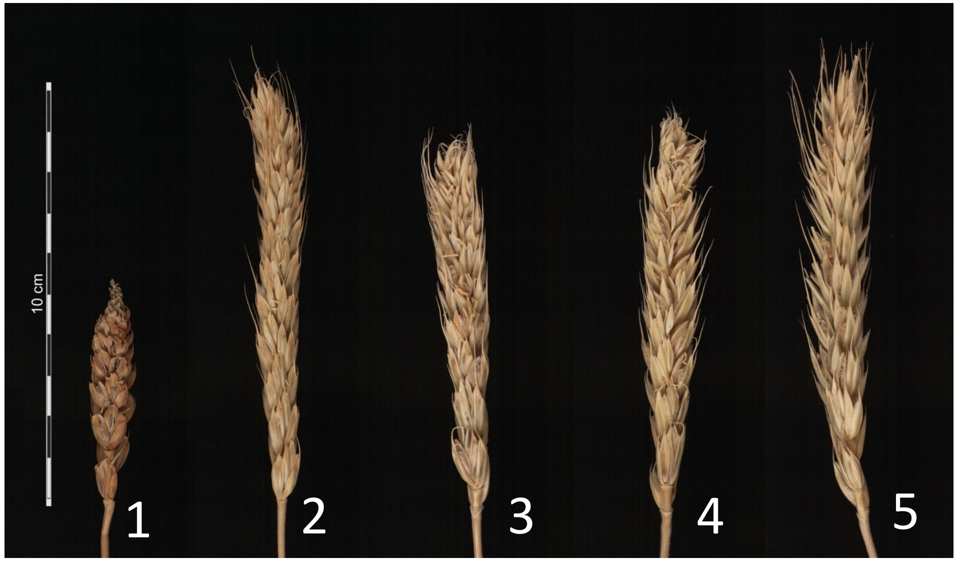

Fig. S8 The spike phenotypes of maternal wheat deletion line CS 6AL-8, amphihaploid and amphidiploid from cross with rye inbred line L2 with *Eml-R1b* allele:

1. Wheat CS 6AL-8 GBS 200 (2n=42)
2. AH6AL8L2 p.24/1 GBS 299 (n=28)
3. AD6AL8L2 p.21 GBS 294 (2n=56)
4. AD6AL8L2 p 13/2 GBS 287 (2n=56)
5. AD6AL8L2 p. 1/4 GBS 274 (2n=56)

Normalized read coverage in 5 Mb bins along the wheat and rye genomes (Chinese Spring V1.0 and Lo7 V1.0 assembly)

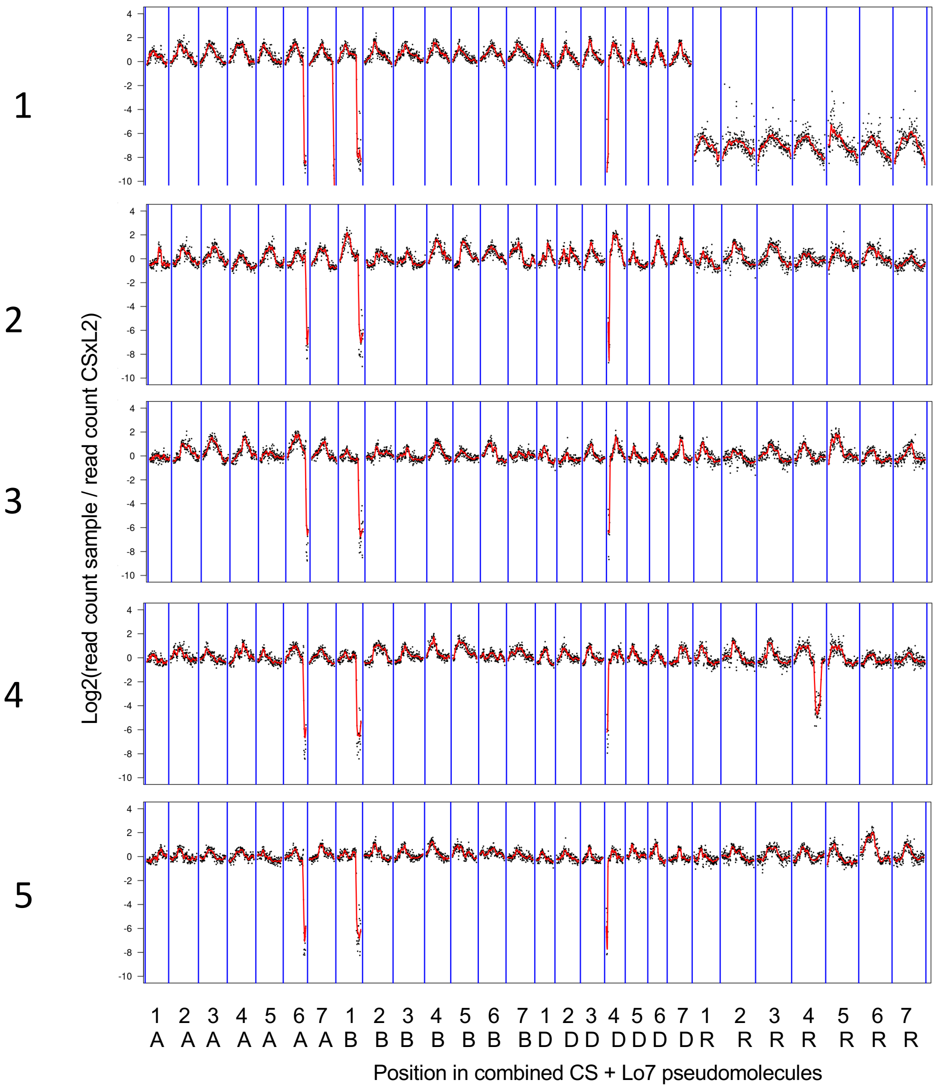

Fig. S8: Spike morphology and normalized read coverage in 5 Mb bins along the wheat and rye genomes (CS V1.0 and Lo7 V1.0 reference assemblies, respectively) of maternal wheat deletion line CS 6AL-8, amphihaploid and amphidiploids from cross 6AL-8 x L2: 1) CS 6AL-8 GBS 200, 2) AH6AL8L2 p.24/1tce GBS 299tce, 3) AD6AL8L2 p.21 GBS 294, 4) AD6AL8L2 p 13/2 GBS 287, 5) AD6AL8L2 p.1/4 GBS 274.
