# Supplementary material for: Major chromosome rearrangements in intergeneric wheat × rye hybrids in compatible and incompatible crosses detected by GBS read coverage analysis
Source: Sci Rep. 2024 May 14;14:11010. doi: 10.1038/s41598-024-61622-1 (PMC11094192; doi:10.1038/s41598-024-61622-1)
Supplement: Supplementary file 10 — Supplementary Information 10. [file 41598_2024_61622_MOESM10_ESM.docx]

Table S2: Reorganization of wheat and rye genomes in intergeneric hybrids from incompatible cross between CS and inbred rye line L2.

| Number of GBS probe | ChrN/  GNP | Reorganization in genome | | | | Chromosome formula in plant |
| --- | --- | --- | --- | --- | --- | --- |
|  |  | A | B | D | R |  |
| ADL2 p. 93 | | | | | | |
| 26 tc | 54/2 | **N6A** |  | *M7D* | M7R | 52(II)+7D(I)+7R(I) |
| 27 tc | 53/0 | **N6A** | *4B*(3) | *M7D* | M7R | 48(II)+4B(III)+7D(I)  +7R(I) |
| 28 tc | 53/25 | **N6A** |  | *M7D* |  | 52(II)+7D(I) |
| 29 | 56*/0 | Dt6AS |  | *Dt7DL* |  | 52(II)+Dt6AL+Dt7DL |
| 30 | 54/4 | *M3A M5A*;Dt6AS | *M2B* | *7DL(2)* |  | 48(II)+Dt6AS+3A(I) +5A(I) +2B(I)+Dt7DL |
| 31 | 54/25 | Dt6AS | *M4B* | *7DL(2)* |  | 50(II)+Dt6AS+  +4B(I)+Dt7DL |
| 32 | 56*/17 | Dt6AS |  | *7DL(2)* |  | 52(II)+Dt6AS+Dt7DL |
| 33 | 54/54 | **N6A** |  | *7DL(2)* | 5R+5RS | 50(II)+Dt7DL+5R+5RS |
| 34 | 53/47 | **N6A** |  | *7DL(2)* | M5R | 50(II)+Dt7DL+5R |
| 35 | 55/1 | Dt6AS |  | *7DL(2)* | 5R+5RS | 50(II)+Dt6AS+Dt7DL +5R+5RS |
| ADL2 p.96/4 | | | | | | |
| 36 | 52/0 | *N5A*; **N6A** |  |  |  | 52(II) |
| 37 | 51/67 | *N5A*; **N6A** |  |  | M3R | 50(II)+3R(I) |
| 38 | 52/40 | *N5A*; **N6A** |  |  | del 5RS | 52(II) |
| 39 | 54/14 | **N6A** |  |  |  | 54(II) |
| 40 | 54/56 | **N6A** |  |  |  | 54(II) |
| 41 | 52/139 | *N5A*; **N6A** |  |  |  | 52(II) |
| 42 | 54/31 | **N6A** |  | *7D+7DS* | 1R+1RL | 50(II)+7D+7DS  +1R+1RL |
| ADL2 p. 139/22 | | | | | | |
| 43 | 56*/240 | Dt6AS | *Dt5BL; Dt7BS* |  |  | 50(II)+Dt6AS+Dt5BL  +Dt7BS |
| 44 | 56*/152 | Dt6AS | *Dt5BL; 7B+7BS* |  |  | 50(II)+Dt6AS+Dt5BL +7B+7BL |
| 45 | 56*/134 | Dt6AS | *Dt5BL* |  |  | 52(II)+Dt6AS+Dt5BL |
| 46 | 56*/187 | Dt6AS | *Dt5BL; Dt7BS* |  |  | 50(II)+Dt6AS+Dt5BL  +Dt7BS |
| 47 | 56*/238 | Dt6AS | *Dt5BL* |  |  | 52(II)+Dt6AS+Dt5BL |
| 48 | 56*/265 | Dt6AS | *Dt5BL; Dt7BS* |  |  | 50(II)+Dt6AS+Dt5BL  +Dt7BS |
| ADL2 p. 229/2 | | | | | | |
| 49 | 54/209 |  |  |  | **N6R** | 54(II) |
| 50 | 55/108 |  |  |  | **M6R** | 54(II)+6R(I) |
| 51 | 56*/51 |  |  |  | Dt6RS | 54(II)+Dt6RS |
| 52 | 54/169 |  |  |  | **N6R** | 54(II) |
| 53 | 55/88 |  |  |  | **M6R** | 54(II)+6R(I) |

| Number of GBS probe | ChrN/  GNP | Reorganization in genome (take place) | | | | Chromosome formula in plant |
| --- | --- | --- | --- | --- | --- | --- |
|  |  | A | B | D | R |  |
| ADL2 p. 233/1 | | | | | | |
| 54 | 55/18 | *del 4AL*;  **del 6AL** | *M4B* |  |  | 54(II)+4B(I) |
| 55 | 56*/64 | *del 4AL*;  **del 6AL** |  |  | 3R+3RS | 54(II)+3R+3RS |
| 56 | 56*/88 | *del 4AL*;  **del 6AL** |  |  | del 6RS | 56(II) |
| 57 | 53/1 | *del 4AL*;  **del 6AL** | *M3B* |  | **N6R** | 52(II)+3B(I) |
| 58 | 54/14 | **del 6AL** |  |  | **N6R** | 54(II) |
| ADL2 p. 250/1 | | | | | | |
| 59 | 42/526 |  |  |  | - all rye genome | 42(II) (AABBDD) |
| 61 | 51/52 | *M5A*; **N6A** |  | *M2D; N7D* | M4R | 48(II)+5A(I)+2D(I)  +4R(I) |
| ADL2 p. 264 | | | | | | |
| 62 | 53/49 |  | *del 1B* | *M2D* | **N6R** | 52(II)+2D(I) |
| 63 | 55/204 |  | *del 1B* |  | **M6R** | 54(II)+6R(I) |
| 64 | 53/18 |  | *del 1B* |  | N1R; M2R | 52(II)+2R(I) |
| 65 | 56*/301 |  | *del 1B* |  |  | 56(II) |
| 66 | 54/161 |  | *del 1B; M1B* |  | **M6R** | 52(II)+1B(1)+6R(I) |
| 67 | 54/2 |  | *del 1B* | M5D | M7R | 52(II)+5D(I)+7R(I) |
| 68 | 54/115 |  | *del 1B;* |  | **M6R**; M7R | 52(II)+6R(I)+7R(I) |
| 69 | 53/5 |  | *del 1B; M6B* |  | **M6R**; M7R | 50(II)+6B(I)+6R(I) +7R(I) |
| ADL2 p. 270/6 | | | | | | |
| 70 | 55/61 | Dt6AS; *7A+7AS* | *4B+4BS* |  |  | 50(II)+6AS+7A+7AS +4B+4BS |
| 71 | 56*/25 | Dt6AS; *Dt7AS* |  |  | del 2R; del5RS | 52(II)+Dt6AS+Dt7AS |
| 72 | 55/249 | Dt6AS; *7A+7AS* |  |  | 4R+4RS? M5R? | 48(II)+Dt6AS+7A+7AS +4R+4RS+5R(I) |
| 73 | 56*/263 | Dt6AS; *7A+7AS* |  |  | del 2R; Dt5RL | 50(II)+Dt6AS+7A+7AS  +Dt5RL |
| 74 | 56*/138 | Dt6AS; *7A+7AS* |  |  | del 2R; 5R+5RL? | 48(II)+Dt6AS+7A+7AS +5R+5RL |
| ADL2 p. 245 | | | | | | |
| 21 tc | 56/0 |  |  |  |  | 56 (II) |
| 22 tc | 56/0 |  |  |  |  | 56 (II) |
| 23 tc | 56/0 |  |  |  |  | 56 (II) |
| 24 tc | 56/0 |  |  |  |  | 56 (II) |
| 25 tc | 56/0 |  |  |  |  | 56 (II) |

| Number of GBS probe | ChrN/  GNP | Reorganization in genome (take place) | | | | Chromosome formula in plant |
| --- | --- | --- | --- | --- | --- | --- |
|  |  | **A** | B | D | AAAR |  |
| AHL2 | | | | | | |
| 16 tce | 28/0 |  |  |  |  | 28 (I) |
| 17 tce | 28/0 |  |  |  |  | 28 (I) |
| 18 tce | 28/0 |  |  |  |  | 28 (I) |
| 19 tce | 28/0 |  |  |  |  | 28 (I) |
| 20 tce | 28/0 |  |  |  |  | 28 (I) |
| CS | | | | | | |
| 6 tce | 40/0 | *M3A* | *M2B* |  |  | 38(II)+3A(I)+2B(I) |
| 7 tce | 42/99 |  |  |  |  | 42(II) |
| 8 tce | 42/87 |  | *del 6BS* |  |  | 42(II) |
| 9 tce | 42/92 |  |  |  |  | 42(II) |
| 10 tce | 42/110 |  |  |  |  | 42(II) |
| 75 tce | 41/91 |  | *1B+1BS; M7B* |  |  | 38(II)+1B+1BS+7B(I) |
| 76 tce | 42/66 |  |  |  |  | 42(II) |

GBS – analysis genotyping-by-sequences; N – nullisomic, M – monosomic, Dt – ditelosomic, del – deletion, (I) – univalent, (II) – bivalent, * - suggests the formation of 28 bivalents in meiosis, despite the presence of deletions or the telosomic state of individual pairs of chromosomes; tc – regenerative plants obtained from immature inflorescences of maternal plant via tissue culture; tce - regenerative plants obtained from immature embryos of maternal plant via tissue culture; ChrN – chromosomes number in plant; GNP – grain number per plant. Wheat and rye chromosomes (or arms) carrying incompatible alleles (*Eml-A1* or *Eml-R1b*) are highlighted in bold type. Wheat chromosomes involved in spontaneous intra- and intergenomic translocations highlighted in italic type.
