# Supplementary material for: Major chromosome rearrangements in intergeneric wheat × rye hybrids in compatible and incompatible crosses detected by GBS read coverage analysis
Source: Sci Rep. 2024 May 14;14:11010. doi: 10.1038/s41598-024-61622-1 (PMC11094192; doi:10.1038/s41598-024-61622-1)
Supplement: Supplementary file 11 — Supplementary Information 11. [file 41598_2024_61622_MOESM11_ESM.docx]

Table S3: Spontaneous translocations in cultivated Emmer and hexaploid wheat.

| References | Chromosome in  tetraploid wheat | Chromosome in common wheat | References | |
| --- | --- | --- | --- | --- |
|  | *T. dicoccoides* | *T. aestivum* |  | |
| Badaeva et al., 2007 | 1A – 2A |  |  | |
| Badaeva et al., 2007 | 1A – 2B |  |  | |
| Badaeva et al., 2007 | 2A – **3B^1)^** |  |  | |
| Badaeva et al., 2007 | **2A – 4B** | **2A** – 4D? | Baier et al., 1974 | |
| Badaeva et al., 2007 | 2A – 6B |  |  | |
| Badaeva et al., 2007 | **3A – 5A** |  |  | |
|  |  | **3A** – 7A | Nakata et al., 1993; Ali et al., 1994 | |
| Badaeva et al., 2007 | **3A – 7B** | **3A – 3B** | Schlegel & Schlegel, 1989 | |
|  |  | 4A pericentric inver.? | Nelson et al., 1995; | |
|  |  | 4A – 6B | Gill & Kimber, 1977 | |
|  |  | 5A – **7B** | Liu et al, 1992; | |
| Badaeva et al., 2007 | 6A – 1B |  |  | |
| Badaeva et al., 2007 | **7A – 5B** | **7A** – 7D | Baier et al., 1974;Linde-Laursen,& Larsen, 1974: Lange et al., 1987 | |
| Badaeva et al., 2007 | **1B – 5B** | **1B** – 2D | Schlegel & Schlegel, 1989 | |
| Badaeva et al., 2007 | **2B – 3B** | **2B** – 2D | Lange et al., 1987 | |
| Badaeva et al., 2007 | **3B** – 4B |  |  | |
| Badaeva et al., 2007 | **3B** – 6B |  |  | |
| Badaeva et al., 2007 | **3B**-5B-7B | **3B** – 3D | Schlegel & Schlegel, 1989 | |
| Badaeva et al., 2007 | **3B – 7B** | **3B – 7B** | Baier et al., 1974; Vega & Lagadena, 1983; Ali et al., 1994 | |
| Badaeva et al., 2007 | **4B – 7B** | **4B** – 6B | Nakata et al., 1993; Taketa et al.,1993; Ali et al.,1992,1994 | |
| Badaeva et al., 2007 | **5B** – 6B |  |  | |
|  | **5B – 7B** | **5B – 7B** | Baier et al., 1974; Jewell, 1979; Lange et al., 1987 | |
|  | *T. dicoccum* |  |  | |
| Badaeva et al., 2007 | 2A - 6A |  |  | |
| Badaeva et al., 2007 | 5A – 6A |  |  | |
|  |  | **4A - 1B** | Zhong & Yao, 1993 | |
| Badaeva et al., 2007 | **4A – 4B** | 4A-6A-7B | Schlegel & Schlegel, 1989 | |
|  |  | 4A-5A-7B | Liu et al, 1992; | |
| Badaeva et al., 2007 | **7A – 5B** |  |  | |
| Badaeva et al., 2007 | **3B – 4B** |  |  | |
| Badaeva et al., 2007 | 3B – 6B |  |  | |
| Badaeva et al., 2007 | **4B – 6B** |  |  | |
| Badaeva et al., 2007 | 6B – 7B | **6B – 7B** | Schlegel & Schlegel, 1989 | |
|  |  | 6B – 1D | Schlegel & Schlegel, 1989 | |
|  |  | 7B – 2D | Schlegel & Schlegel, 1989 | |
|  |  |  |  | |
| References | Chromosome in  tetraploid wheat | Chromosome in common wheat | References | |
|  | *T. carthlicum* |  |  | |
| Badaeva et al., 2007 | **2A – 4B** |  |  | |
|  | *T. turanicum* |  |  | |
| Badaeva et al., 2007 | **5B – 7B** |  |  | |
|  |  |  |  | |
|  | *T. araraticum* |  |  | |
| Badaeva et al., 1993 | 2A – 6A |  |  | |
| Badaeva et al., 1993 | 3A – 7A |  |  | |
| Badaeva et al., 1993 | 2A – 4G |  |  | |
| Kawahara, 1986* | 2At-4G |  |  | |
| Badaeva et al., 1993 | 2A – 6G |  |  | |
| Badaeva et al., 1993 | 2A – 7G |  |  | |
| Badaeva et al., 1993 | 5A – 1G |  |  | |
| Badaeva et al., 1993 | 5A – 3G |  |  | |
| Kawahara, 1986 | 5At–3G |  |  | |
| Badaeva et al., 1993 | 5A – 6G |  |  | |
| Badaeva et al., 1993 | 5A – 7G |  |  | |
| Kawahara, 1986 | 5At–7G |  |  | |
| Badaeva et al., 1993 | 6A – 4G |  |  | |
| Badaeva et al., 1993 | 7A – 3G |  |  | |
| Badaeva et al., 1993 | 7A – 7G |  |  | |
| Kawahara, 1986; Badaeva et al., 1993 | 1G – 2G |  |  | |
| Badaeva et al., 1993 | 1G – 3G |  |  | |
| Kawahara, 1986; Badaeva et al., 1993 | 1G - 4G |  |  | |
| Kawahara, 1986; Badaeva et al., 1993 | 1G – 5G |  |  | |
| Badaeva et al., 1993 | 2G – 4G |  |  | |
| Kawahara, 1986 | 2G-4G-6G |  |  | |
| Kawahara, 1986; Badaeva et al., 1993 | 2G – 6G |  |  | |
| Badaeva et al., 1993 | 2G – 7G |  |  | |
| Kawahara, 1986; Badaeva et al., 1993 | 3G – 4G |  |  | |
| Kawahara, 1986; Badaeva et al., 1993 | 3G – 6G |  |  | |
| Badaeva et al., 1995 | 3G – 7G |  |  | |
| Kawahara, 1986Badaeva et al., 1993 | 4G – 5G |  |  | |
| Kawahara, 1986; Badaeva et al., 1993 | 4G – 6G |  |  | |
| Kawahara, 1986; Badaeva et al., 1993 | 6G – 7G |  |  | |
| References | Chromosome in  tetraploid wheat | Chromosome in common wheat | | References |
|  | *T. aethiopicum* |  | |  |
| Kawahara & Taketa, 2000; Badaeva et al., 2007 | **2A – 4B** |  | |  |
| Badaeva et al., 2007 | **2A-2B-4B** |  | |  |
| Kawahara & Taketa, 2000 | 5A inversion |  | |  |
| Kawahara & Taketa, 2000; Badaeva et al., 2007 | 1B – 6B |  | |  |
| Kawahara & Taketa, 2000 | **4B-2A-2B ct** |  | |  |
| Kawahara & Taketa, 2000; Badaeva et al., 2007 | 5B – 6B |  | |  |
|  | *T. timopheevii* |  | |  |
| Rodrigues et al., 2000 | 3At – 4At |  | |  |
| Salina et al., 2006 | 4At – 6At |  | |  |
| Rodrigues et al., 2000 | 4At – 4G |  | |  |
| Rodrigues et al., 2000 | 5At – 3G |  | |  |
| Jiang, Gill 1994;  Rodrigues et al., 2000 | 6At – 1G |  | |  |
| Rodrigues et al., 2000 | 1G – 2G |  | |  |
| Jiang, Gill 1994;  Rodrigues et al., 2000 | 1G – 4G |  | |  |
| Rodrigues et al., 2000 | 5G – 6G |  | |  |
|  | Emmer genome |  | |  |
| Kawahara , 1997 | 1A – 5A |  | |  |
| Kawahara, 1997 | 2A - 2B |  | |  |
| Kawahara, 1997 | 2A – 4B |  | |  |
| Naranjo et al., 1988 | **4A-5A-7B ct** | **4A-5A-7B** | | Liu et al., 1992; |
| Kawahara, 1997 | 7A – 5B |  | |  |
| Kawahara, 1997 | 2B – 3B |  | |  |
| Kawahara, 1997 | 3B – 4B |  | |  |
| Kawahara, 1997 | 5B – 7B |  | |  |
| Kawahara, 1997 | 6B – 7B |  | |  |

1) – Wheat chromosomes involved in spontaneous intra- and intergenomic translocations both in cultivated Emmer and in hexaploid wheat, highlighted in bold type. - Kawahara T., 1986 - cited in: Badaeva, E.D., Gill, B.S., Badaev, N.S., Kawahara, T., Filatenko, A.A. Chromosomal rearrangements and the process of intraspecific diversity in *Triticum araraticum*. In: Proceedings of the 8^th^ International Wheat Genetics Symposium, Beijing, China, 20-25 July, V.1:293-298, (1993).

References:

1. Ali, A.M., Nakata, N., Tomita, M., Yasumuro, Y. Detection and identification Japanese common wheat varieties. Japan. J. Breed. 42: 573-582, <https://doi.org/10.1270/jsbbs1951.42.573> (1992).
2. Ali, A.M., Nakata, N., Tomita, M., Yasumuro, Y. Identification and breeding significance of translocated chromosomes in a japanese common wheat variety Eshimashinriki. Breeding Science 44: 391-396, <https://doi.org/10.1270/jsbbs1951.44.391>, (1994).
3. Badaeva, E.D., Gill, B.S., Badaev, N.S., Kawahara, T., Filatenko, A.A. Chromosomal rearrangements and the process of intraspecific diversity in *Triticum araraticum*. In: Proceedings of the 8th International Wheat Genetics Symposium, Beijing, China, 20-25 July, V.1:293-298, (1993).
4. [Badaeva](https://cdnsciencepub.com/doi/abs/10.1139/g95-128#con1), E.D., [Jiang](https://cdnsciencepub.com/doi/abs/10.1139/g95-128#con2), J., [Gill](https://cdnsciencepub.com/doi/abs/10.1139/g95-128#con3), B.S. Detection of intergenomic translocations with centromeric and noncentromeric breakpoints in *Triticum araraticum*: mechanism of origin and adaptive significance. [*Genome*](https://cdnsciencepub.com/journal/gen), **38**:976-981 <https://doi.org/10.1139/g95-128> (1995).
5. Badaeva, E.D. *et al.* Chromosomal rearrangements in wheat: their types and distribution. *Genome* **50**: 907-926, <https://doi.org/10.1139/G07-072> (2007).
6. Baier, A.S., Zeller, F.J., Fischbeck, G. Identification of three chromosomal interchanges in common wheat, Triticum aestivum L. *Can. J. Genet. Cytol.,* **16**: 349-354, <https://doi.org/10.1139/g74-039> (1974).
7. Naranjo, T., Roca, A., Goicoechea, P.G., Giraldes, R. Chromosome structure of common wheat: genome reassignment of chromosomes 4A and 4B. In: Miller TE, Koebner RMDs (eds) Proc 7^th^ Int Wheat Genet Symposium, Bath Press, Avon, pp 115-120, (1988).
8. Jewell, D.C. Recognition of alien material and chromosome rearrangements in wheat using N-banding. In: Proc. 5^th^ Intern. Wheat Genet Symp., New Delhi (India), 23-28 February 1978, V.1-2, pp.1208-1212 (1979).
9. Jiang, J., Gill, B.S. Different species-specific chromosome translocation in *Triticum timopheevii* and *T. turgidum* support diphyletic origin of polyploidy wheats. *Chromosome Res* **2**: 59-64, <https://doi.org/10.1007/BF01539455> (1994).
10. Kawahara, T. Screening of spontaneous translocations in cultivated Emmer wheat. Wheat Information Service, **85**: 45-46, (1997).
11. Kawahara, T., Taketa, S. Fixation of translocation 2A·4B infers the monophyletic origin of Ethiopian tetraploid wheat. *Theor Appl Genet* **101**: 705–710, <https://doi.org/10.1007/s001220051534> (2000).
12. Linde-Laursen, I., Larsen, J. The use of double-monotelodisomics to identify translocations in Triticum aestivum. *Hereditas* **78**: 245-250 <https://doi.org/10.1111/j.1601-5223.1974.tb01445.x> (1974).
13. Rodriguez, S., Perera, E., Maestra, B., Diez, M., Naranjo, T. Chromosome structure of *Triticum timopheevii* relative to *T. turgidum.* *Genome* **43**: 923-930, <https://doi.org/10.1139/g00-062> (2000).
14. Salina, E.A. *et al.* Wheat genome structure: translocations during the course of polyploidization. *Funct Integr Genomics* **6:** 71–80, <https://doi.org/10.1007/s10142-005-0001-4> (2006).
15. Zhong S.B., Yao, J.X. Cytogenetic studies on a reciprocal chromosome translocation between 1B and 4A in common wheat. In: Proceedings of the 8^th^ International Wheat Genetics Symposium, Beijing, China, 20-25 July, V.1:279-281(1993).
16. Vega, C., Lacadena, J.R. Identification of two chromosomal interchanges in cv. Canaleja of common wheat, *Triticum aestivum* L. *Euphytica* **32,** 485–491, <https://doi.org/10.1007/BF00021459>(1983).
17. Lange, W. *et al.* Cytogenetic analysis of structural rearrangements in three varieties of common wheat, *Triticum aestivum*. *Theoret. Appl. Genetics* **73,** 635–645, <https://doi.org/10.1007/BF00260770> (1987).
18. Nakata, N., Ali, A.M., Tomita, M., Yasimuro, Y. Translocated chromosomes detected in japanese common wheat varieties. In: Proceedings of the 8^th^ International Wheat Genetics Symposium, Beijing, China, 20-25 July, V.1:283-286, (1993).
19. Liu, C.J. *et al.* Nonhomoeologous translocations between group 4, 5 and 7 chromosomes within wheat and rye. *Theoret. Appl. Genetics* **83,** 305–312, <https://doi.org/10.1007/BF00224276> (1992).
20. Nelson, J.C. Molecular mapping of wheat: Major genes and rearrangements in homoeologous groups 4, 5 and 7*. Genetics* **141**: 721-731, <https://doi.org/10.1093/genetics/141.2.721> (1995).
21. Schlegel, G., Schlegel, R. A compendium of reciprocal inter-varietal translocations in hexaploid wheat. *Die Kulturpflanze* **37,** 163–176 <https://doi.org/10.1007/BF01984613> (1989).
