# Supplementary material for: Major chromosome rearrangements in intergeneric wheat × rye hybrids in compatible and incompatible crosses detected by GBS read coverage analysis
Source: Sci Rep. 2024 May 14;14:11010. doi: 10.1038/s41598-024-61622-1 (PMC11094192; doi:10.1038/s41598-024-61622-1)
Supplement: Supplementary file 12 — Supplementary Information 12. [file 41598_2024_61622_MOESM12_ESM.docx]

Table S4: Reorganization of wheat and rye genomes in intergeneric hybrids ADL2 generation R_3_.

| Number of GBS probe  R_3_ | ChrN/  GNP | Reorganization in genome | | | | Chromosome formula in plant | Spike fertility |
| --- | --- | --- | --- | --- | --- | --- | --- |
|  |  | A | B | D | R |  |  |
| ♀ ADL2 p. 233/1 GBS 54 R_2_ | | | | | | | |
| ♀ 54 R_2_ | 55/18 | *del 4AL*; **del 6AL** | *M4B* |  |  | 54(II)+4B(I) | s-fer^1)^ |
| 153 | 56*/18 | *del 4AL*; **del 6AL** | *Dt4BS* |  |  | 54(II)+Dt4BS | s-fer |
| 154 | 54/117 | M1A; *del4AL*; **del 6AL** |  |  | M3R | 52(II)+1A(I)? +3R(I) | fer^2)^ |
| 155 | 54/0 | *del4AL*; **del 6AL** | *N4B* |  |  | 54(II) | st^3)^ |
| 156 | 54/1 | *del4AL*; M5A; **del 6AL** | *M4B* |  |  | 52(II)+5A(1) +4B(I) | st |
| 157 | 55/1 | *del4AL*; **del 6AL** | *M4B* |  |  | 54(II)+4B(I) | st |
| 158 | 54/0 | *del4AL*; **del 6AL** | *N4B* |  |  | 54(II) | st |
| 159 | 56*/146 | *del4AL*; **del 6AL** |  |  |  | 56(II) | fer |
| 160 | 55/33 | *del4AL*; **del 6AL** |  |  | M2R | 54(II)+2R(I) | s-fer |
| 161 | 55/2 |  | *M4B* |  |  | 54(II)+4B(I) | st |
| 162 | 54/0 | *del4AL*; **del 6AL** | *N4B* |  |  | 54(II) | st |
| ♀ADL2 p. 264/3 GBS 64 R_2_ | | | | | | | |
| ♀ 64 R_2_ | 53/18 |  | *del 1B* |  | N1R; M2R | 52(II)+2R(I) | s-fer |
| 163 | 52/0 |  | *del 1B* |  | N1R; N2R | 52(II) | sklred^4)^, st |
| 164 | 52/0 |  | *del 1B* |  | N1R; N2R | 52(II) | sklred, st |
| 165 | 52/0 |  | *del 1B* |  | N1R; N2R | 52(II) | sklred, st |
| 166 | 53/8 |  | *del 1B* |  | N1R; M2R | 52(II)+2R(I) | s-fer |
| 167 | 52/0 |  | *del 1B* |  | N1R; N2R | 52(II) | sklred, st |
| 168 | 52/0 |  | *del 1B* |  | N1R; N2R | 52(II) | sklred, st |
| 169 | 52/0 |  | *del 1B* |  | N1R; N2R | 52(II) | sklred. st |
| 170 | 52/0 |  | *del 1B* |  | N1R; N2R | 52(II) | sklred, st |
| 171 | 53/6 |  | *del 1B* |  | N1R; M2R | 52(II)+2R(I) | s-fer |
| 172 | 52/11 |  | *del 1B* |  | N1R; M2R; M3R | 50(II)+2R(I)  +3R(I) | s-fer |
| 173 | 52/0 |  | *del 1B* |  | N1R; N2R | 52(II) | sklred, st |

| Number of GBS probe  R_3_ | ChrN/  GNP | Reorganization in genome | | | | Chromosome formula in plant | Spike fertility |
| --- | --- | --- | --- | --- | --- | --- | --- |
|  |  | A | B | D | R |  |  |
| ♀ ADL2 p. 264/6 GBS 67 R_2_ | | | | | | | |
| ♀ 67 R_2_ | 54/2 |  | *del 1B* | M5D | M7R | 52(II)+5D(I)+7R(I) | s-fer |
| 174 | 55/0 |  | *del 1B; 4B(3)* | M5D | M7R | 50(II)+4B(II+I)+5D(I)+7R(I) | st |
| 175 | 54/0 |  | *del 1B* | M5D | M7R | 52(II)+5D(I)+7R(I) | st |
| ♀ ADL2 p. 264/4 GBS 65 R_2_ | | | | | | | |
| ♀ 65 R_2_ | 56*/301 |  | *del 1B* |  |  | 56 | fer |
| 176 | 55/649 |  | *del 1B* |  | M3R | 54(II)+3R(I) | fer |
| 177 | 56*/513 |  | *del 1B* |  |  | 56 | fer |
| 178 | 56*/810 |  | *del 1B* |  |  | 56 | fer |
| 179 | 56*/584 |  | *del 1B* |  |  | 56 | fer |
| 180 | 54/155 |  | *del 1B* | M5D; | M1R | 52(II)+5D(I)+1R(I) | fer |
| 181 | 56*/370 |  | *del 1B* |  |  | 56 | fer |
| 182 | 56*/636 |  | *del 1B* |  |  | 56 | fer |
| 183 | 54/231 |  | *del 1B* |  | **M6R**; M7R | 52(II)+**6R(I)**+7R(I) | fer |
| 184 | 55/111 |  | *del 1B*; dup *5BS*? | M1D |  | 54(II)+1D(I) | fer |
| *185* | *56*/97* |  | *del 1B* |  |  | *56* | *fer* |

GBS – analysis genotyping-by-sequences; N – nullisomic, M – monosomic, Dt – ditelosomic, del – deletion, (I) – univalent, (II) – bivalent, * - suggests the formation of 28 bivalents in meiosis, despite the presence of deletions or the telosomic state of individual pairs of chromosomes. ChrN – chromosomes number in plant; GNP – grain number per plant. 1) – semi-fertile (s-fer); 2) fertile plant (fer); 3) - sterile plant (st); 4) – spikelet reduction (sklred); R_2_ – generation of maternal plants. Wheat and rye chromosomes (or arms) carrying incompatible alleles (*Eml-A1* or *Eml-R1b*) are highlighted in bold type. Wheat chromosomes involved in spontaneous intra- and intergenomic translocations highlighted in italic type.
