# Supplementary material for: Major chromosome rearrangements in intergeneric wheat × rye hybrids in compatible and incompatible crosses detected by GBS read coverage analysis
Source: Sci Rep. 2024 May 14;14:11010. doi: 10.1038/s41598-024-61622-1 (PMC11094192; doi:10.1038/s41598-024-61622-1)
Supplement: Supplementary file 13 — Supplementary Information 13. [file 41598_2024_61622_MOESM13_ESM.docx]

Table S5: Reorganization of wheat and rye genomes in AD31L2 intergeneric hybrids from compatible cross CS nulli-tetrasomic N6AT6D line with inbred rye line L2 generation R_0_.

| Number of GBS probe | GNP/ GNMS | Reorganization in genome | | | | | | Chromosome formula in plant |
| --- | --- | --- | --- | --- | --- | --- | --- | --- |
|  |  | A | B | D | | R | |  |
| AD31L2 seed set more 70% | | | | | | | | |
| 211 | 40/32 | **N6A**^1)^ |  | T6D | |  | | 56(II) |
| 213 | 199/37 | **N6A** |  | T6D | |  | | 56(II) |
| 229 | 145/38 | **N6A** |  | T6D | |  | | 56(II) |
| 236 | 266/37 | **N6A** |  | T6D | |  | | 56(II) |
| AD31L2 seed set 50-69% | | | | | | | | |
| 212 | 120/30 | **N6A** |  | T6D | |  | | 56(II) |
| 218 | 284/38 | **N6A** |  | T6D | |  | | 56(II) |
| 221 | 28/16 | **N6A** |  | T6D; del 6DS | |  | | 56(II) |
| 226 | 273/30 | **N6A** |  | T6D | |  | | 56(II) |
| 227 | 77/38 | **N6A** |  | T6D; del 6DS | |  | | 56(II) |
| 234 | 67/21 | **N6A** |  | T6D; del 6DS | |  | | 56(II) |
| 235 | 32/18 | **N6A** |  | T6D; del 6DS | |  | | 56(II) |
| 237 | 18/18 | **N6A** |  | T6D; del 6DS | |  | | 56(II) |
| 239 | 131/22 | **N6A** |  | T6D | |  | | 56(II) |
| 244 | 115/38 | **N6A** |  | T6D | |  | | 56(II) |
| 246 | 201/35 | **N6A** |  | T6D | |  | | 56(II) |
| 252 | 31/15 | **N6A** |  | T6D; del6DS | |  | | 56(II)* ^2)^ |
| 254 | 490/20 | **N6A** |  | T6D | |  | | 56(II) |
| 255 | 302/46 | **N6A** |  | T6D del 6DS | |  | | 56(II) |
| 258 | 171/27 | **N6A** |  | T6D | | del1RL;  del 5RL | | 56(II)* |
| 260 | 587/36 | **N6A** |  | T6D | |  | | 56(II) |
| 264 | 186/17 | **N6A** |  | T6D; del 6DS | | Dt1RL; M2R | | 52(II)+Dt1RL  +2R(1)* |
| AD31L2 seed set 30-49% | | | | | | | | |
| 215 | 27/13 | **N6A** |  | T6D; del 6DS | |  | | 56(II) |
| 217 | 47/22 | **N6A** |  | T6D; del 6DS | |  | | 56(II) |
| 222 | 189/26 | **N6A** | *7B+7BS*^3)^ | T6D | |  | | 54(II)+7B+7BS |
| 224 | 18/12 | **N6A** |  | T6D; del 6DS | |  | | 56(II)* |
| 231 | 57/20 | **N6A** |  | T6D | |  | | 56(II)* |
| 232 | 153/27 | **N6A** |  | T6D | |  | | 56(II) |
| 238 | 153/14 | **N6A** |  | T6D | |  | | 56(II) |
| 242 | 211/21 | **N6A** |  | T6D | |  | | 56(II)* |
| 245 | 14/7 | **N6A** | *1B+1BS* | T6D | |  | | 54(II)+1B+1BS* |
| 253 | 32/8 | **N6A** |  | T6D | |  | | 56(II)* |
| 256 | 94/25 | **N6A** |  | T6D; del 6DS | |  | | 56(II)* |
| 261 | 559/26 | **N6A** |  | T6D | |  | | 56(II) |
| 263 | 204/25 | **N6A** |  | T6D | |  | | 56(II) |
| AD31L2 seed set 10-29% | | | | | | | | |
| 214 | 5/5 | **N6A** | *5B+5BS* | T6D; del 6DS | |  | | 54(II)+5B+5BS* |
| 238 | 15/15 | **N6A** |  | T6D | |  | | 56(II) |
| 248 | 15/11 | **N6A** |  | T6D; del 6DS | |  | | 56(II) |
| 249 | 20/8 | **N6A** | *5B+5BS* | T6D | |  | | 54(II)+5B+5BS* |
| 251 | 9/5 | **N6A** | *4B+4BL* | T6D; del 6DS | |  | | 54(II)+4B+4BL* |
| 262 | 9/4 | **N6A** |  | N2D; T6D | |  | | 54(II) |
| Number of GBS probe | GNP/ GNMS | Reorganization in genome | | | | | | Chromosome formula in plant |
|  |  | A | B | | D | | R |  |
| AD31L2 seed set <10% | | | | | | | | |
| 223 | 11/3 | **N6A** | *7B+7BS* | | T6D; del 6DS; | |  | 54(II)+7B+7BS* |
| 233 | 1/0 | **N6A** |  | | T6D; del 6DS | |  | 56(II)* |
| 241 | 2/2 | **N6A** |  | | T6D | |  | 56(II) |
| AD31L2 sterile | | | | | | | | |
| 250 | 0/0 | **N6A** | *M4B* | | T6D; | |  | 54(II)+4B(I)* |
| ♀N6AT6D | | | | | | | | |
| 199 | - | **N6A** |  | | T6D; | |  | 42(II) |
| AH31L2 | | | | | | | | |
| 206 s^4)^ | 0/0 | **-6A** |  | | +6D; del 6DS | |  | 26(I)+6D(II): del 6DS |
| 207 s | 0/0 | **-6A** |  | | +6D; del 6DS | |  | 26(I)+6D(II):  del 6DS |
| 208 s | 0/0 | **-6A** |  | | +6D | |  | 26(I)+6D(II) |
| 209 s | 0/0 | **-6A** |  | | +6D; del 6DS; del 7DL | |  | 26(I)+6D(II):  del 6DS; del 7DL |
| 210 s | 0/0 | **-6A** |  | | +6D; del 6DS | |  | 26(I)+6D(II):  del 6DS |
| 216tce^5)^ | 0/0 | **-6A** |  | | +6D;del 6DS; | |  | 26(I)+6D(II):  del 6DS* |
| 220tce | 0/0 | **-6A** |  | | +6D; del 6DS | |  | 26(I)+6D(II):  del 6DS |
| 225tce | 0/0 | **-6A** |  | | +6D; | |  | 26(I)+6D(II) |
| 228tce | 0/0 | **-6A** |  | | +6D; | |  | 26(I)+6D(II) |
| 230tce | 0/0 | **-6A** |  | | +6D; | |  | 26(I)+6D(II) |
| 240tce | 0/0 | **-6A** |  | | +6D; | |  | 26(I)+6D(II) |
| 243tce | 0/0 | **-6A** |  | | +6D | |  | 26(I)+6D(II) |
| 247tce | 0/0 | **-6A** |  | | +6D; | |  | 26(I)+6D(II) |
| 259tce | 0/0 | **-6A** |  | | +6D; | |  | 26(I)+6D(II) |

GBS – analysis genotyping-by-sequences; N – nullisomic, M – monosomic, T – tetrasomic, del – deletion, (I) – univalent, (II) – bivalent; 1) Chromosomes of the maternal plants, missing after direct selection, highlighted in bold and italic. 2) * - plant has multiple indels in chromosome structure; 3) - wheat chromosomes involved in spontaneous intra- and intergenomic translocations highlighted bold and italic; 4) – s - amphihaploid obtained from hybrid seed without tissue culture method; 5) - tce – amphihaploid obtained from immature hybrid wheat-rye embryos via tissue culture. GNP- grain number per plant; GNMS – grain number in main spike of the plant.
