# Supplementary material for: Major chromosome rearrangements in intergeneric wheat × rye hybrids in compatible and incompatible crosses detected by GBS read coverage analysis
Source: Sci Rep. 2024 May 14;14:11010. doi: 10.1038/s41598-024-61622-1 (PMC11094192; doi:10.1038/s41598-024-61622-1)
Supplement: Supplementary file 14 — Supplementary Information 14. [file 41598_2024_61622_MOESM14_ESM.docx]

Table S6: Haplotypes of fertile amphidiploids obtained in compatible crosses of CS nulli-tetrasomic N6AT6D and CS 6AL-8 deletion lines with inbred rye line L2.

| Haplotype | Reorganization in genome | | | | Chromosome formula in plant |
| --- | --- | --- | --- | --- | --- |
|  | A | B | D | R |  |
| 1 | N6A |  | T6D |  | 56(II) |
| 2 | N6A |  | T6D;  del 6DS |  | 56(II) |
| 3 | del 6AL; | del 1BL | del 4DS |  | 56(II) |
| 4 | del 6AL; | del 1BL | del 4DS | del 4RL | 56(II) |
| 5 | del 6AL; | del 1BL | del 4DS |  | 56(II)* |
| 6a | del 6AL;  del 7AL | del 1BL | del 4DS |  | 56(II) |
| 6b | del 6AL;  del 7AL | del 1BL | del 4DS | del 2RS | 56(II) |

N – nullisomic, T – tetrasomic, del – deletion, (II) – bivalent; * - plant has multiple indels in chromosome structure.
