# Supplementary material for: Major chromosome rearrangements in intergeneric wheat × rye hybrids in compatible and incompatible crosses detected by GBS read coverage analysis
Source: Sci Rep. 2024 May 14;14:11010. doi: 10.1038/s41598-024-61622-1 (PMC11094192; doi:10.1038/s41598-024-61622-1)
Supplement: Supplementary file 15 — Supplementary Information 15. [file 41598_2024_61622_MOESM15_ESM.docx]

Table S7: Characteristics of main spike of amphidiploids from compatible crosses.

| Cross | Fertility  (%) | Haplotype | Number of plants | Characteristics of main spike | | | | | GNP | TGW (g) | Number spikes per plant |
| --- | --- | --- | --- | --- | --- | --- | --- | --- | --- | --- | --- |
|  |  |  |  | Length (cm) | Number of spikelets | GN | Seed set (%) | Weight of grains (g) |  |  |  |
| AD31L2 | N6A/T6D x L2 | | | | | | | | | | |
|  | >70 | **1** | **4** | **10.1** | **23.0** | **36.0** | **82.2** | **1.59** | **162.5** | **44.1** | **7.0** |
|  | 50-69 | 1 | 9 | 10.8 | 26.1 | 31.6 | 63.1 | 1.28 | 286.1 | 40.6 | 12.2 |
|  |  | 2 | 9 | 9.5 | 22.4 | 24.0 | 55.1 | 0.95 | 101.3 | 39.1 | 8.3 |
|  |  | **mean value** | **18*** | **10.1** | **24.3** | **27.8** | **59.1** | **1.11** | **193.7** | **39.8** | **10.3** |
|  | 30-49 | 1 | 5 | 10.0 | 26.8 | 25.6 | 41.7 | 1.1 | 186.2 | 42.2 | 10.2 |
|  |  | 2 | 7 | 9.0 | 19.3 | 17.3 | 41.0 | 0.70 | 94.6 | 38.7 | 6.1 |
|  |  | **mean value** | **12*** | **9.3** | **22.4** | **20.8** | **41.3** | **0.87** | **132.8** | **40.2** | **7.8** |
|  | 10-29 | 2 | **6*** | **8.6** | **20.0** | **8.0** | **22.4** | **0.27** | **12.2** | **35.4** | **4.2** |
|  | <10 | 1 | 1 | 10.5 | 25.0 | 2.0 | 5.9 | 0.08 | 2.0 | 40.0 | 8.0 |
|  |  | 2 | 2 | 9.5 | 20.0 | 1.5 | 3.0 | 0.06 | 6.0 | 30.7 | 9.0 |
|  |  | **mean value** | **3*** | **9.8** | **21.7** | **1.7** | **3.9** | **0.06** | **4.7** | **33.8** | **8.7** |
| AD6AL-8L2 | 6AL-8 x L2 | | | | | | | | | | |
|  | >70 | 3 | **1** | **9.3** | **26.0** | **34.0** | **72.5** | **1.57** | **347.0** | **46.2** | **29.0** |
|  | 50-69 | 3 | 2 | 9.9 | 25.0 | 29.5 | 52.1 | 1.20 | 445.0 | 40.2 | 26.5 |
|  |  | 4 | 1 | 10.5 | 27.0 | 29.0 | 50.0 | 1.20 | 649.0 | 41.4 | 36.0 |
|  |  | 5 | 2 | 9.9 | 27.0 | 26.0 | 54.9 | 0.95 | 428.5 | 36.4 | 25.0 |
|  |  | 6b | 1 | 9.4 | 17.0 | 20.0 | 53.1 | 0.68 | 24.0 | 33.8 | 2.0 |
|  |  | **mean value** | **6*** | **9.8** | **24.7** | **26.7** | **53.0** | **1.02** | **403.7** | **38.1** | **23.5** |
|  | 30-49 | 3 | 6 | 10.0 | 25.5 | 18.3 | 38.4 | 0.68 | 140.7 | 36.8 | 14.2 |
|  |  | 5 | 1 | 8.5 | 22.0 | 11.0 | 39.3 | 0.36 | 11.0 | 32.7 | 3.0 |
|  |  | 6a | 1 | 9.0 | 29.0 | 10.0 | 30.0 | 0.24 | 43.0 | 24.2 | 10.0 |
|  |  | **mean value** | **8*** | **9.7** | **25.3** | **16.4** | **37.4** | **0.59** | **112.3** | **34.6** | **12.3** |
|  | 10-29 | 3 | 1 | 12.0 | 27.0 | 13.0 | 16.7 | 0.4 | 69.0 | 30.6 | 18.0 |
|  |  | 4 | 1 | 10.3 | 33.0 | 9.0 | 19.6 | 0.34 | 9.0 | 37.8 | 26.0 |
|  |  | 5 | 1 | 11.2 | 25.0 | 10.0 | 15.6 | 0.30 | 51.0 | 30.0 | 12.0 |
|  |  | **mean value** | **3*** | **11.2** | **28.3** | **10.7** | **17.3** | **0.35** | **43.0** | **30.2** | **18.7** |
|  | <10 | 3 | 1 | 11.5 | 28.0 | 4.0 | 6.5 | 0.10 | 25.0 | 25.2 | 16.0 |
|  |  | 5 | 4 | 9.3 | 22.3 | 2.0 | 3.3 | 0.07 | 8.3 | 28.6 | 10.6 |
|  |  | 6b | 1 | 14.0 | 27.0 | 0.0 | 0.0 | 0.0 | 1.0 | 34.1 | 18.0 |
|  |  | **mean value** | **6*** | **11.6** | **26.3** | **2.5** | **5.0** | **0.06** | **13.0** | **32.8** | **12.8** |

* - the total number of analyzed plants in a given fertility class; GN – grain number in main spike; GNP – grain number per plant; TGW - thousand-grain weight in grams.
