# Supplementary material for: Major chromosome rearrangements in intergeneric wheat × rye hybrids in compatible and incompatible crosses detected by GBS read coverage analysis
Source: Sci Rep. 2024 May 14;14:11010. doi: 10.1038/s41598-024-61622-1 (PMC11094192; doi:10.1038/s41598-024-61622-1)
Supplement: Supplementary file 16 — Supplementary Information 16. [file 41598_2024_61622_MOESM16_ESM.docx]

Table S8: Reorganization of wheat and rye genomes in AD6AL-8L2 intergeneric hybrids from compatible cross CS 6AL-8 deletion line with inbred rye line L2 generation R_0_.

| Number of GBS probe | GNP/ GNMS | Reorganization in genome | | | | Chromosome formula in plant |
| --- | --- | --- | --- | --- | --- | --- |
|  |  | A | B | D | R |  |
| AD6AL-8L2 seed set more 70% | | | | | | |
| 294 | 347/34 | **del 6AL**^1)^ | *del 1BL*^2)^ | *del 4DS* |  | 56(II) |
| AD6AL-8L2 seed set 50-69% | | | | | | |
| 273 | 333/22 | **del 6AL** | *del 1BL* | *del 4DS* |  | 56(II) |
| 274 | 557/37 | **del 6AL** | *del 1BL* | *del 4DS* |  | 56(II) |
| 275 | 24/20 | **del 6AL**; *del 7AL* | *del 1BL* | *del 4DS* | del 2RS | 56(II) |
| 276 | 580/24 | **del 6AL** | *del 1BL* | *del 4DS* |  | 56(II) |
| 284 | 277/28 | **del 6AL** | *del 1BL* | *del 4DS* |  | 56(II)* ^3)^ |
| 287 | 649/29 | **del 6AL** | *del 1BL* | *del 4DS* | del 4RL | 56(II) |
| AD6AL-8L2 seed set 30-49% | | | | | | |
| 272 | 216/23 | **del 6AL** | *del 1BL* | *del 4DS* |  | 56(II) |
| 301 | 169/17 | **del 6AL** | *del 1BL* | *del 4DS* |  | 56(II)* |
| 278 | 11/11 | **del 6AL** | *del 1BL* | *del 4DS* |  | 56(II)* |
| 281 | 102/11 | **del 6AL** | *del 1BL* | *del 4DS* |  | 56(II) |
| 282 | 147/26 | **del 6AL** | *del 1BL* | *del 4DS* |  | 56(II) |
| 285 | 47/20 | **del 6AL** | *del 1BL* | *del 4DS* |  | 56(II) |
| 289 | 43/10 | **del 6AL**; *del 7AL* | *del 1BL* | *del 4DS* |  | 56(II) |
| 272 | 216/23 | **del 6AL** | *del 1BL* | *del 4DS* |  | 56(II) |
| AD6AL-8L2 seed set 10-29% | | | | | | |
| 283 | 51/10 | **del 6AL** | *del 1BL* | *del 4DS* |  | 56(II)* |
| 286 | 9/9 | **del 6AL** | *del 1BL* | *del 4DS* | del 4RL | 56(II) |
| 298 | 69/13 | **del 6AL** | *del 1BL* | *del 4DS* |  | 56(II) |
| AD6AL-8L2 seed set <10% | | | | | | |
| 279 | 23/3 | **del 6AL** | *del 1BL* | *del 4DS* |  | 56(II)* |
| 290 | 1/0 | **del 6AL** | *del 1BL* | *del 4DS* |  | 56(II)* |
| 295 | 25/4 | **del 6AL** | *del 1BL* | *del 4DS* |  | 56(II) |
| 304 | 3/3 | **del 6AL** | *del 1BL* | *del 4DS* |  | 56(II) |
| CS deletion line 6AL-8 | | | | | | |
| 200 | - | **del 6AL**; *del 7AL* | *del 1B* | *del 4DS* |  | 42(II) |
| AH6AL-8L2 | | | | | | |
| 277tce^4)^ | 0/0 | **del 6AL**; *del 7AL* | *del 1BL* | *del 4DS* |  | 28(I) |
| 288tce | 0/0 | **del 6AL**; *del 7AL* | *del 1BL* | *del 4DS* |  | 28(I) |
| 291tce | 0/0 | **del 6AL** | *del 1BL* | *del 4DS* |  | 28(I) |
| 292tce | 0/0 | **del 6AL** | *del 1BL* | *del 4DS* |  | 28(I) |
| 296tce | 0/0 | **del 6AL** | *del 1BL* | *del 4DS* |  | 28(I) |
| 299tce | 0/0 | **del 6AL** | *del 1BL* | *del 4DS* |  | 28(I) |
| 300tce | 0/0 | **del 6AL** | *del 1BL* | *del 4DS* |  | 28(I)* |
| 302tce | 0/0 | **del 6AL** | *del 1BL* | *del 4DS* |  | 28(I)* |
| 303tce | 0/0 | del 6AL | *del 1BL* | *del 4DS* |  | 28(I)* |
| 305tce | 0/0 | **del 6AL**; *del 7AL* | *del 1BL* | *del 4DS* | del 2RS | 28(I)* |

GBS – analysis genotyping-by-sequences; (I) – univalent, (II) – bivalent; del – deletion, 1) - the part of the mother plant chromosome missing after direct selection is highlighted in bold and italic. 2) - wheat chromosomes involved in spontaneous intra- and intergenomic translocations highlighted bold and italic. 3) * - plant has multiple indels in chromosome structure; 4) - tce – amphihaploid obtained from immature hybrid wheat-rye embryos via tissue culture. GNP- grain number per plant; GNMS – grain number in main spike of the plant.
